# Blood pressure lowering in isolated diastolic hypertension and cardiovascular risk: an individual patient data meta-analysis

**DOI:** 10.1093/eurheartj/ehaf962

**Published:** 2025-12-12

**Authors:** Zeinab Bidel, Milad Nazarzadeh, Dexter Canoy, John William McEvoy, Rusitanmujiang Maimaitiaili, John Chalmers, Koon K Teo, Carl J Pepine, Barry R Davis, Kazem Rahimi, Blood Pressure Lowering Treatment Trialists’ Collaboration, Amanda Adler, Larry Agodoa, Ale Algra, Folkert W Asselbergs, Nigel S Beckett, Eivind Berge, Henry Black, Eric Boersma, Frank P J Brouwers, Morris Brown, Jasper J Brugts, Christopher J Bulpitt, Robert P Byington, William C Cushman, Jeffrey Cutler, Richard B Devereaux, Jamie P Dwyer, Ray Estacio, Robert Fagard, Kim Fox, Tsuguya Fukui, Ajay K Gupta, Rury R Holman, Yutaka Imai, Masao Ishii, Stevo Julius, Yoshihiko Kanno, Sverre E Kjeldsen, John Kostis, Kizuku Kuramoto, Jan Lanke, Edmund Lewis, Julia B Lewis, Michel Lievre, Lars H Lindholm, Stephan Lueders, Stephen MacMahon, Giuseppe Mancia, Masunori Matsuzaki, Maria H Mehlum, Steven Nissen, Hiroshi Ogawa, Toshio Ogihara, Takayoshi Ohkubo, Christopher R Palmer, Anushka Patel, Marc Allan Pfeffer, Bertram Pitt, Neil R Poulter, Hiromi Rakugi, Gianpaolo Reboldi, Christopher Reid, Giuseppe Remuzzi, Piero Ruggenenti, Takao Saruta, Joachim Schrader, Robert Schrier, Peter Sever, Peter Sleight, Jan A Staessen, Hiromichi Suzuki, Lutgarde Thijs, Kenji Ueshima, Seiji Umemoto, Wiek H van Gilst, Paolo Verdecchia, Kristian Wachtell, Paul Whelton, Lindon Wing, Mark Woodward, Yoshiki Yui, Salim Yusuf, Alberto Zanchetti, Zhen-Yu Zhang, Craig Anderson, Colin Baigent, Barry Morton Brenner, Rory Collins, Dick de Zeeuw, Jacobus Lubsen, Ettore Malacco, Bruce Neal, Vlado Perkovic, Anthony Rodgers, Peter Rothwell, Gholamreza Salimi-Khorshidi, Johan Sundström, Fiona Turnbull, Giancarlo Viberti, Jiguang Wang

**Affiliations:** Deep Medicine, Nuffield Department of Women's & Reproductive Health, Medical Sciences Division, University of Oxford, Second Floor, Osney One Building, Osney Mead, Oxford, OX2 0EW, UK; Deep Medicine, Nuffield Department of Women's & Reproductive Health, Medical Sciences Division, University of Oxford, Second Floor, Osney One Building, Osney Mead, Oxford, OX2 0EW, UK; Population Health Sciences Institute, Newcastle University, Newcastle upon Tyne, UK; Department of Cardiology, University of Galway School of Medicine, Galway, Ireland; Department of Cardiology, Shanghai Tenth People’s Hospital, Tongji University, Shanghai, China; The George Institute for Global Health, University of New South Wales, Sydney, Australia; Population Health Research Institute, Hamilton Health Sciences, McMaster University, Hamilton, Ontario, Canada; College of Medicine, University of Florida, Gainesville, FL, USA; School of Public Health, The University of Texas, Houston, TX, USA; Deep Medicine, Nuffield Department of Women's & Reproductive Health, Medical Sciences Division, University of Oxford, Second Floor, Osney One Building, Osney Mead, Oxford, OX2 0EW, UK

**Keywords:** Major cardiovascular diseases, Isolated diastolic hypertension, Blood pressure-lowering treatment, Individual participant-level data

## Abstract

**Background and Aims:**

Blood pressure (BP) lowering reduces cardiovascular disease (CVD) risk; however, the benefits of treating patients with normal systolic BP but elevated diastolic BP remain uncertain.

**Methods:**

Data from 51 randomized controlled trials were pooled to compare BP-lowering effects in participants with and without isolated diastolic hypertension (IDH), defined as systolic BP < 130 mmHg and diastolic BP ≥ 80 mmHg. Treatment effects were stratified across baseline diastolic BP categories (range < 60 to ≥90 mmHg) among individuals with baseline systolic BP < 130 mmHg. Fixed-effect one-stage individual participant data meta-analyses were used, and Cox proportional hazard models, stratified by trial, were applied to analyse the data.

**Results:**

Among 358 325 participants, 15 845 (4.4%) had IDH. At a median follow-up of 4.2 years, a 5 mmHg reduction in systolic BP reduced the risk of major cardiovascular events similarly in individuals with IDH [hazard ratio 0.91; 95% confidence interval (CI) 0.82–1.01] and those without IDH (hazard ratio 0.90; 95% CI 0.89–0.92; *P* for interaction = 1.00). Analyses by baseline diastolic BP showed no evidence of heterogeneity in treatment effects among individuals with baseline systolic BP < 130 mmHg (*P* for interaction = .26). Relative treatment effects were not statistically different by CVD history, age, prior medication use, and BP measurement methods.

**Conclusions:**

The study found no evidence to suggest that pharmacological BP-lowering therapy in individuals with IDH is less or more effective than in those without IDH. Relative risk reductions also did not diminish in those with lower diastolic BP, down to <60 mmHg at baseline. No meaningful differences across various clinical phenotypes were detected.

## Structured Graphical Abstract

**Structured Graphical Abstract:**
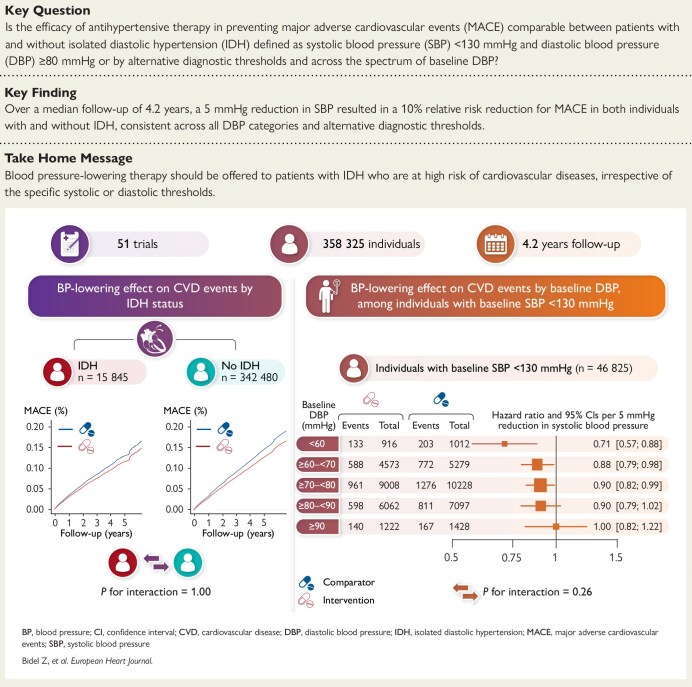
This figure illustrates that pharmacological blood pressure-lowering therapy reduces major cardiovascular events equally in individuals with and without isolated diastolic hypertension, with consistent benefits across all baseline diastolic blood pressure categories.

**See the editorial comment for this article ‘Should systolic pressure be lowered in isolated diastolic hypertension?’, by P. Verdecchia *et al*., https://doi.org/10.1093/eurheartj/ehaf950.**

## Introduction

Elevated blood pressure (BP) is a major global contributor to cardiovascular diseases (CVD) and associated mortality.^1–3^ Although pharmacological BP lowering is a well-established strategy for mitigating CVD risk in a wide range of at-risk groups,^4,5^ its preventive effect in patients with elevated diastolic BP (DBP) remains a topic of ongoing debate.^6–9^ This debate is particularly relevant for managing isolated diastolic hypertension (IDH) in the context of current evidence-based guidelines recommending more intensive BP treatment targets.^10^

Isolated diastolic hypertension has been traditionally defined as a systolic BP (SBP) < 140 mmHg and a DBP ≥ 90 mmHg.^11^ However, in recent years and with recommendations for tighter BP control, the American College of Cardiology/American Heart Association (ACC/AHA) changed the definition of IDH to SBP at <130 mmHg and DBP at ≥80 mmHg.^12^ Although BP lowering has been recommended in this patient group, this has been based on a low level of evidence.^10^ To date, no randomized trials have been conducted specifically in patients with IDH. Furthermore, evidence from epidemiological studies investigating the association between IDH and CVD outcomes has been inconsistent, challenging the case of BP-lowering therapy in this patient group.^13–15^

Given the strong correlation between SBP and DBP, the fraction of patients meeting the definition of IDH is expected to be relatively small. Additionally, patients with IDH tend to be younger with a lower predicted risk of CVD. These characteristics render the conduct of new prospective trials in IDH challenging. In the absence of such trials and given the known limitations of non-randomized studies, the analysis of data from existing large-scale trials led by the Blood Pressure Lowering Treatment Trialists’ Collaboration (BPLTTC) can provide more reliable answers to this question.^4,5,16^ In this study, we utilized the extensive BPLTTC dataset to investigate the effects of pharmacological BP-lowering treatments in individuals with and without IDH, as well as across various DBP categories.

## Methods

### Study setting, study design, and eligibility criteria

We performed an individual participant data (IPD) meta-analysis using data from the BPLTTC. The BPLTTC represents a consortium of principal investigators from leading clinical trials dedicated to the evaluation of pharmacological interventions for BP lowering. This collaborative effort is coordinated by the University of Oxford, located in Oxford, UK. The Deep Medicine research group at the University of Oxford conducted all analyses centrally, using individual-level data collected from each eligible trial. According to the most recent update (August 2024), the collaboration includes data from 52 randomized controlled trials.

The methodological framework and design specifics of the BPLTTC have been extensively reported in previous publications.^4,5,16^ Eligible trials were identified through comprehensive literature search and principal investigators and data custodians of the trials were then invited to participate and contribute IPD. In the current study, we included all trials with at least 1000 person-years of follow-up per randomly assigned group that reported data on age, BP levels at randomization and during follow-up, and CVD events. Trials exclusively involving patients with heart failure, short-term interventions, or those conducted in the context of acute myocardial infarction or other acute conditions were excluded. A statistical analysis plan, including pre-specified subgroup analyses (with the null hypothesis of no heterogeneity of effect in subgroups), was developed before the dataset was released for statistical analysis.^17^ This analysis plan was finalized following thorough input from international collaborators and the BPLTTC steering committee. Ethical approval was obtained by the BPLTTC from the Oxford Tropical Research Ethics Committee (OxTREC Reference 545–14). Informed consent had already been obtained from participants in each of the included trials.

The systematic review protocol, including details of the methods and search strategy, was registered in PROSPERO (CRD42018099283) before the review was conducted.

### Treatment and comparison groups

In each trial, treatment and comparator groups were defined according to the trial design, consistent with previous BPLTCC studies.^4,5^ For placebo-controlled trials, the placebo group was designated as the comparator and the active treatment group as the intervention. In head-to-head trials comparing two or more drug classes, the group achieving greater BP reduction was considered the treatment group, while the other was the comparator. For trials comparing BP-lowering strategies, such as intensive vs standard approaches, the intensive group was classified as the treatment and the standard group as the comparator. Detailed information on the comparison groups, participant characteristics, trial designs, and levels of BP reduction has been published previously.^4,5,16,18,19^ We defined IDH status as individuals with a threshold of SBP < 130 mmHg and DBP ≥ 80 mmHg at baseline. This definition was based on four considerations: first, it aligns with the definition of IDH in US guidelines; second, according to the 2024 ESC guidelines, for adults at high cardiovascular risk, consideration may be given to treating IDH (class IIb recommendation-reflecting limited and conflicting evidence but expert consensus favouring intervention) once DBP exceeds the designated threshold; third, most updated guidelines generally recommend on-treatment BP targets of <130/80 mmHg; and fourth, this BP threshold for IDH definition remains controversial.^9^ Sensitivity analysis was also conducted with a more conservative definition of SBP < 140 mmHg and DBP ≥ 90 mmHg.

### Primary and secondary outcomes

The primary outcome was defined as the first occurrence of a major cardiovascular event, including fatal or non-fatal stroke or cerebrovascular disease (both ischaemic and haemorrhagic), fatal or non-fatal ischaemic heart disease, or heart failure resulting in death or hospitalization. The secondary outcomes were all-cause death and each component of the primary outcome. The diagnostic information provided by each trial was used to define the outcomes.

### Statistical analysis

We performed a complete case analysis because the proportion of missing baseline SBP and DBP data was minimal (0.1% for each). Since this rate is substantially below the 5% threshold where imputation is generally recommended, this approach is robust and avoids potential bias from imputation.^20,21^

We conducted an intention-to-treat analysis, categorizing participants according to their original random allocation within each trial. A fixed-effect one-stage IPD meta-analysis was used, applying a unified statistical model to the IPD from all trials simultaneously.^22^ The hazard ratio (HR) and corresponding 95% confidence intervals (CIs) were estimated using a Cox proportional hazards model, stratified by trial. Event rates were estimated using Kaplan–Meier estimates of cumulative incidence and plotted separately for IDH at baseline. The estimates were standardized for a reduction in SBP of 5 mmHg at the trial level, closely approximating the mean reduction achieved across BP-lowering intensity and placebo-controlled trials.^4,16^ An interaction term for IDH status and treatment was incorporated into the model to assess the heterogeneity of effect by baseline IDH status. Detailed methodology for effect size standardization is provided in the Supplementary data online, *Method S1*.

In people with SBP < 130 mmHg at baseline, subgroup analysis was conducted to assess the heterogeneity of treatment effect across baseline DBP categories in 10 mmHg increments, ranging from 60 to 90 mmHg. In individuals with IDH at baseline, we examined treatment effects by history of CVD, baseline age, and prior use of antihypertensive medication. We also performed analysis to explore the interaction between IDH status and treatment, considering trials that utilized specific BP measurement methods, including automated and manual readings. Moreover, we repeated the entire analysis according to the IDH diagnostic criteria of SBP < 140 mmHg and DBP ≥ 90 mmHg and standardized for a reduction in DBP of 3 mmHg instead of SBP.

The likelihood ratio test was used to evaluate the interaction between the treatment and characteristics of interest. To minimize the risk of false-positive results, *P*-values for interaction were adjusted for multiple comparisons using the Hommel method.^17,23^ Statistical significance was defined as *P* < .05 for all analyses. All statistical analyses were done using R (version 4.4.1).

## Results

Of 52 randomized trials, one study was excluded due to the unavailability of time-to-event data (Efficacy of Candesartan on Outcome in Saitama Trial),^24^ resulting in 51 trials being included in this study. Among the 358 325 participants included in the analysis, 15 845 (4.4%) were identified as having IDH at baseline. The baseline characteristics at random allocation are shown in *Table 1*. The mean age was 60.6 years in people with IDH and 65.2 years in those without IDH. Furthermore, the proportion of women was lower among those with IDH, at 30.0%, compared with those without IDH (42.1%). Ischaemic heart disease and atrial fibrillation represented the most and least prevalent comorbidities, respectively, across IDH status. Overall, 74.9% of participants with IDH and 69.9% without IDH had a prior history of non-trial antihypertensive medications. In both groups, the predominant medications were β-blockers, ACE inhibitors, antiplatelets, and lipid-lowering agents (*Table 1*).

**Table 1:** Baseline characteristics of participants stratified by isolated diastolic hypertension status

Characteristics	Isolated diastolic hypertension (15 845)	No isolated diastolic hypertension (342 480)
Age, years	60.6 (9.4)	65.2 (9.6)
Female sex	4747 (30.0)	144 309 (42.1)
Systolic BP, mmHg	122.5 (5.1)	153.8 (20.7)
Diastolic BP, mmHg	84.0 (4.8)	87.5 (12.6)
Body mass index, kg/m^2^	28.5 (5.2)	27.9 (7.4)
Comorbidity		
Peripheral vascular disease	544 (9.3)	12 346 (9.6)
Atrial fibrillation	972 (6.1)	9507 (2.8)
Diabetes	3924 (24.8)	97 590 (28.6)
Chronic kidney disease	2036 (16.6)	57 105 (20.9)
Cerebrovascular disease	2758 (22.0)	48 036 (17.4)
Ischaemic heart disease	7924 (50.1)	112 125 (32.9)
Previous use of non-trial medications		
Diuretic	2159 (19.5)	38 888 (21.9)
α-Blocker	283 (4.4)	6268 (4.6)
β-Blocker	4613 (41.7)	55 383 (29.7)
ACE inhibitor	2973 (31.2)	55 328 (33.9)
Angiotensin II receptor blocker	237 (4.1)	8383 (8.7)
Calcium channel blocker	3143(28.4)	60 915 (32.6)
Any BP-lowering drug	11 672 (74.9)	221 277 (69.9)
Antiplatelet	3134 (59.8)	47 858 (42.5)
Anticoagulant	558 (11.4)	6010 (7.3)
Lipid-lowering treatment	3867 (51.4)	50 600 (35.6)

Data are *n* (%) or mean (standard deviation).

ACE, angiotensin-converting enzyme; BP, blood pressure.

**Figure 1:**
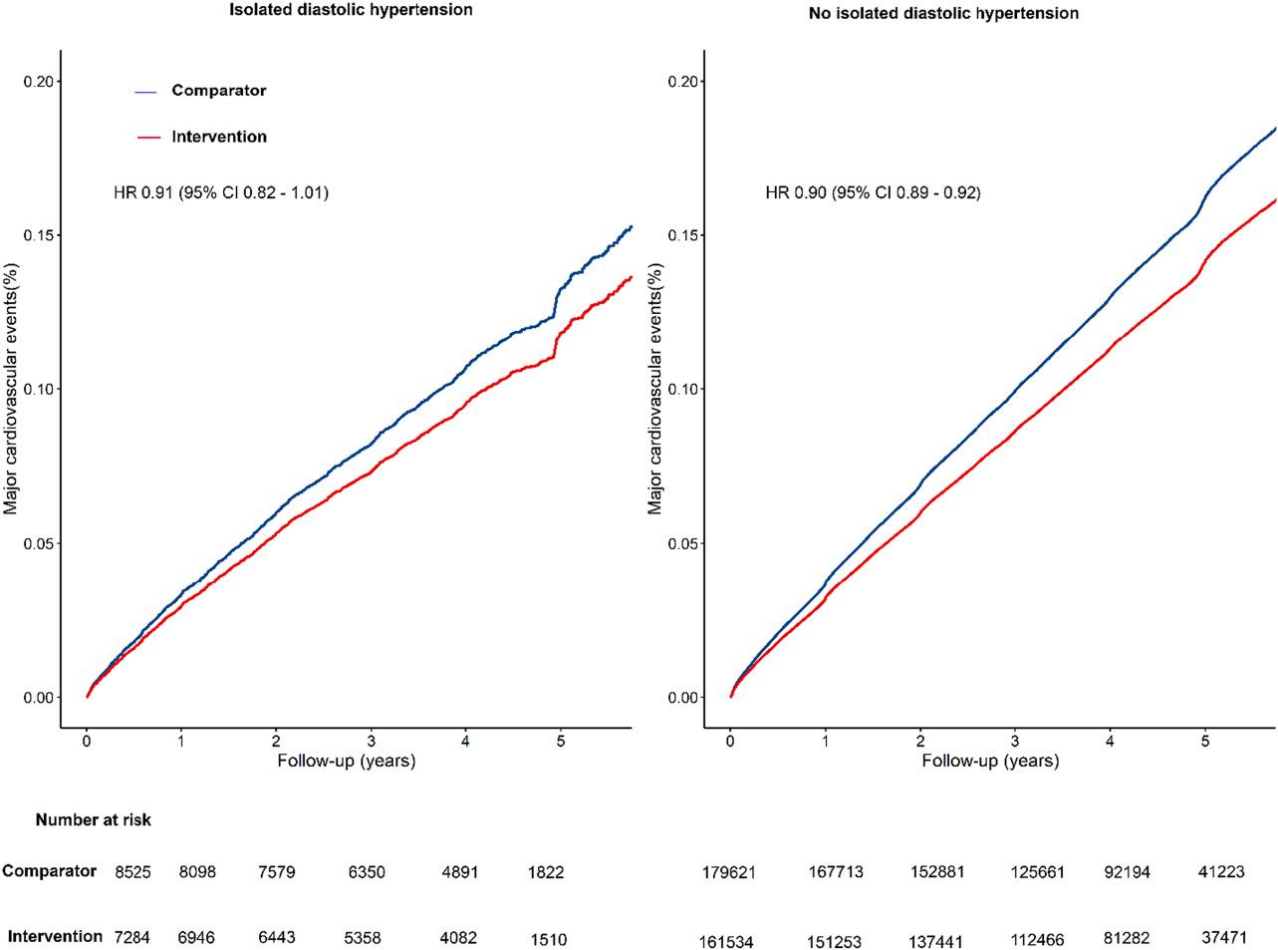
Cumulative incidence of major cardiovascular events per 5 mmHg reduction in systolic blood pressure, by treatment allocation and for isolated diastolic hypertension. Major cardiovascular events are defined as a composition of fatal or non-fatal stroke, fatal or non-fatal myocardial infarction or ischaemic heart disease, or heart failure causing death or requiring hospitalization. IDH, isolated diastolic hypertension; BP, blood pressure

**Figure 2:**
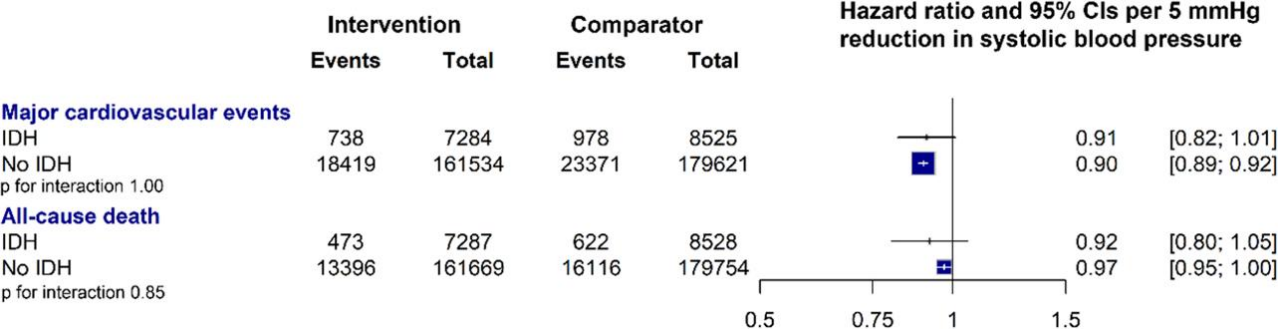
Effect of blood pressure–lowering treatment on major cardiovascular events and deaths, stratified by people with and without isolated diastolic hypertension at baseline. Forest plot shows the hazard ratios and 95% confidence intervals per 5 mmHg systolic blood pressure reduction, separately for each outcome

During a median follow-up of 4.2 years [interquartile range (IQR), 3.2–4.9], there were 43 506 major CVD events. Of these, 1716 occurred in individuals with IDH. Among participants without IDH at baseline, the absolute risk for major CVD event was 130.1 per 1000 (95% CI 128.6–131.7) in the comparator group and 114.0 per 1000 (95% CI 112.5–115.6) in the intervention group. In participants with IDH at baseline, the corresponding rates were 114.7 per 1000 (95% CI 108.1–121.7) in the comparator group and 101.3 per 1000 (95% CI 94.6–108.5) in the intervention group, respectively (*Figure 1*). The HR for the risk of major CVD associated with a 5 mmHg reduction in SBP was 0.91 (95% CI, 0.82–1.01) in people with IDH, and 0.90 (95% CI, 0.89–0.92) in those without IDH (*P* for interaction = 1.00) (*Figures 1* and *2*). Analyses of the treatment effects on the risk of all-cause mortality and on each component of the primary outcome, considered as secondary outcomes stratified by IDH status, were broadly consistent with the results of the primary outcome, with no heterogeneity of effect (*Figures 1* and *2*; Supplementary data online, *Figure S1*).

**Figure 3:**
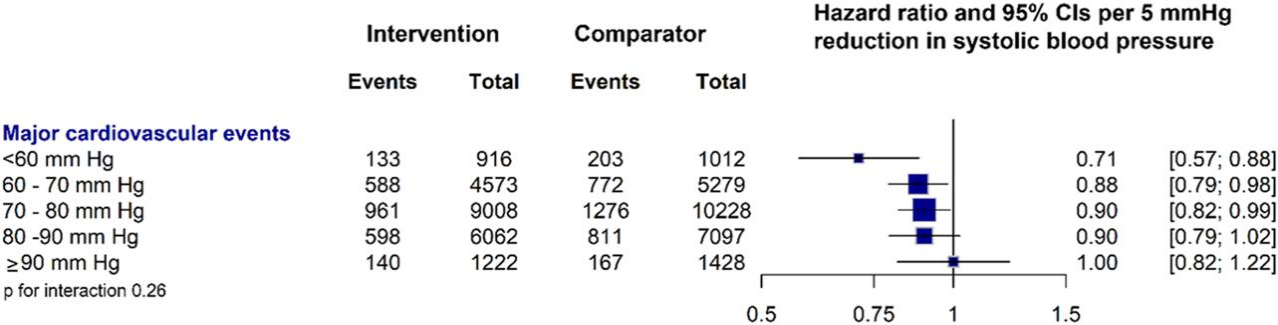
Effect of blood–pressure lowering treatment on major cardiovascular events, stratified by diastolic blood pressure categories at baseline, for people with systolic blood pressure of <130 mmHg at baseline. Forest plot shows the hazard ratios and 95% confidence intervals per 5 mmHg systolic blood pressure reduction. Within each category, the mean and standard deviation (SD) for diastolic blood pressure at baseline were as follows: <60 mmHg [mean 55.28 (SD 3.5)], 60–69 mmHg [mean 64.69 (SD 3.1)], 70–79 mmHg [mean 73.72 (SD 3.1)], 80–89 mmHg [mean 82.29 (SD 2.7)], and >90 mmHg [mean 92.00 (SD 3.3)]

Among individuals with baseline SBP < 130 mmHg, there was no evidence of effect modification by DBP category on major cardiovascular event risk (*P* for interaction = 0.26), nor any attenuation of the relative treatment effect at lower DBP; accordingly, the overall relative treatment effect should be considered the most reliable estimate (*Figure 3*). In stratified analyses of individuals with IDH, we observed no clinically meaningful heterogeneous treatment effects between those with and without prior CVD diseases at baseline (see Supplementary data online, *Figure S2*). Similarly, no evidence of heterogeneity in relative treatment effects was detected across different age categories (all *P* for interaction = 1.00) (see Supplementary data online, *Figure S3*). Although the effect sizes varied slightly among the age categories, the widest CIs were noted in groups with ages older than 75 at baseline. This variation likely reflects the smaller numbers of IDH participants and events in this age group (see Supplementary data online, *Figure S3*). In the analyses stratified by the use of antihypertensive drugs at baseline, the effect sizes were broadly consistent with no heterogeneity in relative effect (see Supplementary data online, *Figure S4*).

In light of the greater variability in DBP than SBP measurement, we conducted analyses by considering only trials that reported BP measurement methods. Among these trials, BP was measured using automated methods in 11 trials and manual methods in 35 trials. The analyses employing the manual method indicated no evidence of heterogeneous treatment effects by IDH status on the risk of major CVD events. However, in trials using the automated method, the smaller number of events led to less precise effect estimates for major cardiovascular events (see Supplementary data online, *Figure S5* and *S6*).

We repeated the main analyses using an alternative definition of IDH as SBP < 140 mmHg and DBP ≥ 90 mmHg.^10^ According to this definition, 12 380 (3.5%) of participants were identified as having IDH. The baseline characteristics were similar to those in the main analysis (see Supplementary data online, *Table S1*). Among individuals with IDH, 1400 CVD events occurred during the follow-up period, with an HR of 0.97 (95% CI, 0.87–1.08). In participants without IDH, there were 42 168 events, with an HR of 0.90 (95% CI, 0.89–0.92) (*P* for interaction = .88) (see Supplementary data online, *Figure S7*). These results did not vary meaningfully across different subgroups, including baseline DBP, history of CVD, age categories, use of antihypertensive medications, and different BP measurement methods (see Supplementary data online, *Figure S8*–*S13*). Risk of bias assessment was conducted using the revised Cochrane risk-of-bias tool^25^ as detailed in our previous study.^4^ Most included studies exhibited low risk of bias across all evaluated domains, with only four studies raising ‘some concerns’ mainly related to the effect of assignment to intervention, which reflects the practical difficulties of maintaining blinding in BP reduction studies. Sensitivity analyses that excluded studies with elevated risk of bias yielded effect sizes that were not different from the primary analysis (see Supplementary data online, *Figure S14*). Analyses standardized by a 3 mmHg reduction in DBP yielded similar findings to the main analyses (see Supplementary data online, *Tables S2–S5*).

## Discussion

In this largest source of randomized evidence of pharmacological BP lowering, we found that in individuals with IDH, the relative effects of BP lowering on CVD risk were similar to those observed in individuals without IDH. No significant evidence was found to suggest a lower or higher effect on the risk of all-cause mortality in those with IDH, compared with those without. Furthermore, across a wide spectrum of baseline DBP levels ranging from <60 to ≥90 mmHg, there was also no strong indication that the relative treatment effect was weaker in those with lower DBP. Although statistical power limited some two-way stratified analyses, we found no evidence of a meaningful difference in relative effects among those with IDH and differing age categories, with or without prior CVD, or with or without prior use of antihypertensives. Alternative definitions of IDH did not change the study findings (*Structured Graphical Abstract*).

The management and implications of IDH have been a topic of debate for decades, with observational studies offering varied perspectives on its association with cardiovascular outcomes. Several observational studies have explored the relationship between IDH or DBP levels and CVD risk, with mixed findings.^13,14,26–28^ A comparison of the definitions of IDH as outlined by the ACC/AHA (SBP < 130 mmHg and DBP ≥ 80 mmHg) and ESC/NICE (SBP < 140 mmHg and DBP ≥ 90 mmHg) guidelines using the UK Biobank cohort showed that while IDH defined by the ACC/AHA was not significantly associated with CVD risk [HR 1.08 (95% CI, 0.98–1.18)], IDH defined by the ESC/NICE was significantly associated with a modest increase in CVD risk [HR 1.15 (95% CI, 1.04–1.29)].^28^ Conversely, other studies, such as that by Strandberg *et al*.,^26^ have reported that IDH alone, without accompanying systolic hypertension, may not significantly increase CVD risk, especially in older populations, with cardiovascular risk less pronounced compared with isolated systolic hypertension or combined hypertension. While findings from these observational studies provide useful insights, clinical implications of treatment effects of BP-lowering drugs could not be derived from these studies, given the non-randomized nature of the comparisons.

Only a few individual trials attempted to provide evidence. The PROGRESS trial, which included 315 participants with IDH for whom 50 cardiovascular events occurred during the follow-up period, showed a 28% relative reduction in risk of CVD. However, the CIs were wide and imprecise, ranging from −29% to 60%.^29^ Given the sample size and number of events in clinical trials addressing IDH, providing robust randomized evidence has been challenging. As a result, clinical decisions regarding BP-lowering treatment for IDH have primarily relied on observational studies or expert opinion.^8^ Our study fills these gaps by providing randomized evidence from a large-scale individual-level data meta-analysis of RCTs, dismissing concerns that antihypertensives in those with IDH might be less effective.

Putting IDH considerations aside, our study also addresses the uncertainty surrounding BP lowering in patients with low baseline DBP. While observational studies have suggested a J-curve relationship between DBP and cardiovascular risk,^30^ our findings demonstrate consistent treatment benefit across all DBP levels, including those below 60 mmHg. These results align with and extend other evidence challenging the causality of this J-curve phenomenon. For instance, SPRINT *post hoc* analyses demonstrated preserved SBP-lowering benefits irrespective of baseline DBP.^31^ Furthermore, Mendelian randomization studies found no evidence for a causal J-curve relationship, instead linking genetically lower DBP to reduced myocardial infarction risk.^32^ Collectively, this evidence suggests the observed J-curve in observational studies likely stems from confounding factors such as arterial stiffness or underlying disease rather than low DBP being directly harmful.^33,34^ Therefore, our findings indicate that a low baseline DBP should not be considered an obstacle to BP-lowering treatment in clinical practice.

The challenge of interpreting IDH’s clinical significance is compounded by its distinct age-related epidemiology, with IDH being more prevalent in individuals younger than 50 years^35^ and decreasing in older age groups.^36^ This pattern aligns with physiological changes where DBP typically peaks in mid-life before declining.^37^ Recent meta-analyses of cohort studies reported that IDH is associated with an increased risk of composite CVD events, with the elevated risk being more pronounced in younger individuals (mean age ≤ 55 years), while the risk was not significant in older populations.^13^ Given the relatively small number of patients with IDH, investigation of treatment effects in subgroups of such patients will be challenging. This also applies to our study that, despite its large size, had limited statistical power to reliably investigate such subgroup effects. Although our analysis did not detect a significant modification of treatment effect by age for individuals with IDH, clinical prudence necessitates individualized assessment, particularly in elderly populations. This approach requires a comprehensive evaluation beyond chronological age alone, incorporating assessment of frailty, multimorbidity, polypharmacy burden, patient preferences, and overall life expectancy.^4,38^ Such holistic consideration ensures that extrapolation of our findings to very elderly or particularly vulnerable patients with IDH is undertaken judiciously, with treatment decisions aligned with patient-specific cardiovascular risk profiles and personalized goals of care.^10,39^ Similarly, we found no evidence to suggest that a history of using antihypertensive drugs or BP measurement methods played a determining role, although the absence of interaction does not provide definitive evidence against the existence of any differential effects.

The findings of our study have important implications for clinical practice, as they significantly reduce uncertainty and could contribute to further simplification of existing recommendations. Our study provides the most comprehensive randomized evidence to date, demonstrating that modest BP lowering confers a consistent protective effect in individuals with IDH, comparable with that observed in individuals without IDH. This beneficial effect persisted across various definitional thresholds for IDH and remained robust for a wide range of adult ages, history of CVD, or prior antihypertensive medication use, although statistical power was limited for certain subgroup analyses. Findings from this study should resolve uncertainties regarding the efficacy of BP-lowering therapy in IDH. Recent debates, highlighted by the 2024 ESC Guidelines, have particularly focused on managing IDH in younger adults who typically exhibit lower absolute cardiovascular risk.^10^ Our findings directly address this issue, demonstrating that the relative cardiovascular risk reduction from BP lowering remains robust even when baseline SBP is within optimal or normotensive range.

Previous research indicates that the rate of antihypertensive treatment is very low among IDH patients. For instance, in the China PEACE study, 3.2% were identified with IDH, of whom 86% did not receive treatment.^40^ In the USA, 8.9% of adults had IDH; however, younger individuals (aged 18–39) show significantly lower levels of awareness and treatment of IDH compared with those aged 40 and older.^41^ Although these studies have not reported the predicted risk of CVD in these patients, it is likely that a fraction of these patients remain untreated despite their elevated risk. Given our findings, clinicians may consider discussing with their patients with IDH and high CVD risk the rationale and potential benefits of initiating BP-lowering therapy, thereby mitigating the likelihood of undertreatment due to previous uncertainties regarding therapeutic efficacy. Rather than depending solely on traditional DBP thresholds (≥90 mmHg) or arbitrary SBP criteria, clinical decisions could incorporate comprehensive cardiovascular risk assessments as recommended by the recent update of the ESC guidelines.^39,42,43^

Some limitations of this study should be taken into account when interpreting the results. Although this is the largest study of randomized comparisons among patients with IDH and across different categories of BP, it has limited statistical power for testing hypotheses of differential effects in some subgroups of patients with IDH. Any strong clinical or biological hypothesis of differing effects would require additional investigations in prospective RCTs. A further limitation of this analysis is the lack of data on treatment-related adverse outcomes such as hypotension, syncope, falls, and acute kidney injury across the included trials, which limits our ability to conduct a reliable pooled assessment of potential harms. Although this study focused on the relative benefits of BP lowering for major cardiovascular outcomes in individuals with IDH, future research should incorporate adverse event reporting to facilitate more comprehensive evaluations of the overall balance of risks and benefits. The inclusion of all potentially eligible studies was not feasible, which represents a common limitation in IPD meta-analyses that rely on voluntary collaboration from trial investigators. However, previous studies using the BPLTTC dataset including extensive sensitivity analysis showed no evidence of data acquisition bias in the BPLTTC dataset.^44^

## Conclusion

These findings indicate that the effect of pharmacological BP-lowering therapy on major CVD events does not differ substantially between individuals with or without IDH, regardless of the specific thresholds used to define the condition, or across different categories of baseline DBP in those with normal SBP. These results suggest the need to reconsider current clinical guidelines, advocating for a more inclusive approach to BP-lowering treatment rather than rigid adherence to the definition of IDH based on BP classifications. For individuals at risk of CVD, including those with IDH, BP-lowering treatment should be recognized as a fundamental strategy for risk prevention, irrespective of baseline BP levels, prior cardiovascular history, or age.

## Supplementary Material

ehaf962_Supplementary_Data

## Declarations

### Data Availability

The Blood Pressure Lowering Treatment Trialists’ Collaboration (BPLTTC) data are governed by the University of Oxford’s research policies and overseen by the BPLTTC Steering Committee. Data cannot be shared with third parties. Requests should be directed to individual trial data custodians. More details are available at https://www.bplttc.org.

### Pre-registered Clinical Trial Number

The systematic review protocol of the BPLTTC, including details of the methods and search strategy, was registered with PROSPERO (www.crd.york.ac.uk/prospero/) (CRD42018099283) before the review was conducted.

## Appendix

Blood Pressure Lowering Treatment Trialists' Collaboration: Amanda Adler, Larry Agodoa, Ale Algra, Folkert W. Asselbergs, Nigel S. Beckett, Eivind Berge, Henry Black, Eric Boersma, Frank P.J. Brouwers, Morris Brown, Jasper J. Brugts, Christopher J. Bulpitt, Robert P. Byington, William C. Cushman, Jeffrey Cutler, Richard B. Devereaux, Jamie P. Dwyer, Ray Estacio, Robert Fagard, Kim Fox, Tsuguya Fukui, Ajay K. Gupta, Rury R. Holman, Yutaka Imai, Masao Ishii, Stevo Julius, Yoshihiko Kanno, Sverre E. Kjeldsen, John Kostis, Kizuku Kuramoto, Jan Lanke, Edmund Lewis, Julia B. Lewis, Michel Lievre, Lars H. Lindholm, Stephan Lueders, Stephen MacMahon, Giuseppe Mancia, Masunori Matsuzaki, Maria H. Mehlum, Steven Nissen, Hiroshi Ogawa, Toshio Ogihara, Takayoshi Ohkubo, Christopher R. Palmer, Anushka Patel, Marc Allan Pfeffer, Bertram Pitt, Neil R. Poulter, Hiromi Rakugi, Gianpaolo Reboldi, Christopher Reid, Giuseppe Remuzzi, Piero Ruggenenti, Takao Saruta, Joachim Schrader, Robert Schrier, Peter Sever, Peter Sleight, Jan A. Staessen, Hiromichi Suzuki, Lutgarde Thijs, Kenji Ueshima, Seiji Umemoto, Wiek H. van Gilst, Paolo Verdecchia, Kristian Wachtell, Paul Whelton, Lindon Wing, Mark Woodward, Yoshiki Yui, Salim Yusuf, Alberto Zanchetti, Zhen-Yu Zhang, Craig Anderson, Colin Baigent, Barry Morton Brenner, Rory Collins, Dick de Zeeuw, Jacobus Lubsen, Ettore Malacco, Bruce Neal, Vlado Perkovic, Anthony Rodgers, Peter Rothwell, Gholamreza Salimi-Khorshidi, Johan Sundström, Fiona Turnbull, Giancarlo Viberti, Jiguang Wang.
