# Supplementary material for: Blood pressure lowering in isolated diastolic hypertension and cardiovascular risk: an individual patient data meta-analysis
Source: Eur Heart J. 2025 Dec 12;47(14):1649–57. doi: 10.1093/eurheartj/ehaf962 (PMC13058465; doi:10.1093/eurheartj/ehaf962)
Supplement: ehaf962_Supplementary_Data [file ehaf962_supplementary_data.docx]

**Supplementary materials**

## **Blood pressure lowering in isolated diastolic hypertension and cardiovascular risk: an individual patient data meta-analysis**

## **The Blood Pressure Lowering Treatment Trialists’ Collaboration**

*Steering Committee*: Kazem Rahimi (Chair), Koon Teo, Barry R Davis, John Chalmers, Carl J Pepine

*Collaborating Trialists*: A Adler (UKPDS [UK Prospective Diabetes Study]), L Agodoa (AASK [African-American Study of Kidney Disease and Hypertension]), A Algra (Dutch TIA Study [Dutch Transient Ischemic Attack Study]), F W Asselbergs (PREVEND-IT [Prevention of Renal and Vascular End-stage Disease Intervention Trial]), N Beckett (HYVET [Hypertension in the Very Elderly Trial]), E Berge (deceased) (VALUE trial [Valsartan Antihypertensive Long-term Use Evaluation trial]), H Black (CONVINCE [Controlled Onset Verapamil Investigation of Cardiovascular End Points]), E Boersma (EUROPA [European trial on reduction Of cardiac events with Perindopril among patients with stable coronary Artery disease]), F P J Brouwers (PREVEND-IT), M Brown (INSIGHT [International Nifedipine GITS Study: Intervention as a Goal in Hypertension]), J Brugts (EUROPA),C J Bulpitt (EWPHE [European Working Party on High Blood Pressure in the Elderly], HYVET), R P Byington (PREVENT [Prospective Randomized Evaluation of the Vascular Effects of Norvasc Trial]), J Chalmers (ADVANCE [Action in Diabetes and Vascular Disease: Preterax and Diamicron MR Controlled Evaluation], PROGRESS [Perindopril protection against recurrent stroke]), W C Cushman (ACCORD [Action to Control Cardiovascular Risk in Diabetes], ALLHAT [Antihypertensive and Lipid-Lowering Treatment to Prevent Heart Attack Trial], SPRINT [Systolic Blood Pressure Intervention Trial]), J Cutler (ALLHAT), B R Davis (ALLHAT), R B Devereaux (LIFE [Losartan Intervention For Endpoint reduction in hypertension]), J P Dwyer (IDNT [Irbesartan Diabetic Nephropathy Trial]), R Estacio (ABCD [Appropriate Blood Pressure Control in Diabetes]), R Fagard (Syst-Eur [SYSTolic Hypertension in EURope]), K Fox (EUROPA), T Fukui (CASE-J [Candesartan Antihypertensive Survival Evaluation in Japan]), A K Gupta (ASCOT-BPLA [AngloScandinavian Cardiac Outcomes Trial—Blood Pressure Lowering Arm]), R R Holman (UKPDS), Y Imai (HOMED-BP [Hypertension Objective Treatment Based on Measurement by Electrical Devices of Blood Pressure]), M Ishii (JMIC-B [Japan Multicenter Investigation for Cardiovascular Diseases-B]), S Julius (VALUE), Y Kanno (E-COST [Efficacy of Candesartan on Outcome in Saitama Trial]), S E Kjeldsen (VALUE, LIFE), J Kostis (SHEP [Systolic Hypertension in the Elderly Program]), K Kuramoto (NICS-EH [National Intervention Cooperative Study in Elderly Hypertensives]), J Lanke (STOP Hypertension-2 [Swedish Trial in Old Patients with Hypertension-2], NORDIL [Nordic Diltiazem]), E Lewis (IDNT), J B Lewis (IDNT), M Lievre (DIABHYCAR [Non-insulin-dependent diabetes, hypertension, microalbuminuria or proteinuria, cardiovascular events, and ramipril study]), L H Lindholm (CAPPP [Captopril Prevention Project], STOP Hypertension-2, NORDIL), S Lueders (MOSES [The Morbidity and Mortality After Stroke, Eprosartan Compared With Nitrendipine for Secondary Prevention]), S MacMahon (ADVANCE, PART-2 [Prevention of Atherosclerosis with Ramipril Trial]), G Mancia (INSIGHT), M Matsuzaki (COPE [The Combination Therapy of Hypertension to Prevent Cardiovascular Events]), M H Mehlum (VALUE), S Nissen (CAMELOT [Comparison of Amlodipine vs Enalapril to Limit Occurrences of Thrombosis]), H Ogawa (HIJ-CREATE [Heart Institute of Japan Candesartan Randomized Trial for Evaluation in Coronary Heart Disease]), T Ogihara (CASE-J, COLM [Combinations of OLMesartan], COPE), T Ohkubo (HOMED-BP), C R Palmer (INSIGHT), A Patel (ADVANCE), C J Pepine (INVEST [International Verapamil SR-Trandolapril Study]), M A Pfeffer (PEACE [Prevention of Events With Angiotensin- Converting Enzyme Inhibition]), B Pitt (PREVENT), N R Poulter (ASCOT), H Rakugi (CASE-J, VALISH [Valsartan in Elderly Isolated Systolic Hypertension Study]), G Reboldi (Cardio-Sis [CARDIOvascolari del Controllo della Pressione Arteriosa SIStolica]), C Reid (ANBP2 [The Second Australian National Blood Pressure Study]), G Remuzzi (BENEDICT [BErgamo NEphrologic DIabetes Complications Trial]), P Ruggenenti (BENEDICT), T Saruta (CASE-J), J Schrader (MOSES), R Schrier (deceased) (ABCD), P Sever (ASCOT-BPLA), P Sleight (deceased; CONVINCE, HOPE [Heart Outcomes Prevention Evaluation], ONTARGET [Ongoing Telmisartan Alone and in Combination with Ramipril Global Endpoint Trial], TRANSCEND [Telmisartan Randomised AssessmeNt Study in ACE iNtolerant subjects with cardiovascular Disease]), J A Staessen (Syst-Eur), H Suzuki (E-COST), L Thijs (Syst-Eur), K Ueshima (CASE-J, VALISH), S Umemoto (COPE), W H van Gilst (PREVEND-IT), P Verdecchia (Cardio-Sis), K Wachtell (LIFE), P Whelton (SPRINT), L Wing (ANBP2), M Woodward (ADVANCE, PROGRESS), Y Yui (JMIC-B), S Yusuf (HOPE, ONTARGET, TRANSCEND), A Zanchetti (deceased; ELSA [European Lacidipine Study on Atherosclerosis], VHAS [Verapamil in Hypertension and Atherosclerosis Study]), and Z Y Zhang (Syst-Eur).

*Other members*: C Anderson, C Baigent, B M Brenner, R Collins, D de Zeeuw, J Lubsen, E Malacco, B Neal, V Perkovic, A Rodgers, P Rothwell, G Salimi-Khorshidi, J Sundström, F Turnbull, G Viberti, and J Wang.

**Method S1: Statistical standardisation approach**

Rationale for Effect Size Standardization:

Standardization of effect sizes is appropriate when the objective is to pool the effects of blood (BP) pressure-lowering treatments and express the effect for a fixed level of BP reduction.^1,2^ This approach is essential for such analyses because the magnitude of BP reduction varies across trials. Standardization facilitates adjustment for this heterogeneity by assigning greater weight to trials with larger BP reductions, particularly when the hypothesis focuses on assessing the BP-mediated effect.

In practical terms, this implies that all else being equal, trials with minimal BP reductions between treatment arms are assigned proportionately lower weights than they would be in the absence of standardization. Another advantage of standardization is that it permits the inclusion of a wide range of BP-lowering trials without necessitating an arbitrary threshold for trial-level achieved BP reduction. For instance, in head-to-head trials comparing one drug with another, the achieved reduction is often modest. Rather than excluding these trials (e.g., with 1 or 2 mm Hg BP reduction), standardization enables their inclusion in the analysis, thereby enhancing statistical power while assigning lower weights to account for the smaller achieved BP reduction.

To estimate the effect size of treatment per a fixed amount of blood pressure reduction, it is therefore essential to standardize effect sizes to a predefined and clinically meaningful blood pressure level. Without this standardization, the estimated effect size (e.g., hazard ratio) would lack scale, and the interpretation of relative risk reduction would have unclear clinical implications.

Statistical Methodology for Standardization

For standardization, first, for each trial, the average achieved BP reduction was estimated using a linear mixed-effects model, with further methodological details reported elsewhere.^3^ This value (i.e., achieved BP reduction) was subsequently treated as the trial-level blood pressure reduction for each trial participant and incorporated into the dataset as a new variable for analysis. A stratified Cox model was then fitted to estimate the hazard ratio for the treatment effect, adjusted for this trial-level BP reduction. Finally, the relative effect size was rescaled to correspond to a 5 mm Hg reduction in systolic BP and a 3 mm Hg reduction in diastolic BP, respectively.

Detailed Statistical Implementation

For clarity, we illustrate below the three steps for estimating the effects of systolic BP lowering treatment on primary outcomes by isolated diastolic hypertension (IDH) status at baseline, with the results shown in Figure 2 of the main paper. The other analyses follow the same approach.

Step 1: Model Specification

First, we fitted the Cox model (Model #1), adjusted for systolic BP reduction at the trial level. The value for systolic BP reduction for each trial was derived from our previous study.

The following model was fitted:

Model #1: treatment + delta + (treatment × delta) + (treatment × IDH) + (treatment × delta × IDH)

Where:

•"treatment" is a binary variable for treatment allocation (0 = comparator, 1 = intervention)

•"delta" is systolic blood pressure reduction at trial level

•"IDH" is isolated diastolic hypertension status at baseline (0 = non-IDH at baseline, 1 = IDH at baseline)

Step 2: Parameter Extraction

At this stage, the following regression coefficients were extracted from the model summary and variance-covariance matrix:

•b_treatment arm = regression coefficient for treatment arm

•b_delta = regression coefficient for systolic blood pressure reduction at trial level

•b_treatment arm:delta = regression coefficient for interaction between treatment arm and systolic blood pressure reduction at trial level

•b_comparator arm:IDH = regression coefficient for interaction between comparator arm and IDH status at baseline

•b_treatment arm:IDH = regression coefficient for interaction between treatment arm and IDH status at baseline

•b_comparator arm:delta:IDH = regression coefficient for interaction between comparator arm, systolic blood pressure reduction at trial level and IDH status at baseline

•b_treatment arm:delta:IDH = regression coefficient for interaction between treatment arm, systolic blood pressure reduction at trial level and IDH status at baseline

Step 3: Rescaling to 5 mm Hg Reduction

Up to this point, the model had been adjusted for systolic BP reduction at the trial level. This means that the model accounts for variations in BP reduction across trials. We then rescaled the hazard ratio to a 5 mm Hg reduction, for people with and without IDH at baseline. From the Cox model, we multiplied appropriate terms in the prediction model by 5 to estimate the hazard ratio per 5 mm Hg reduction for each category of IDH at baseline:

Model #2: Standardized log hazard ratio per 5 mm Hg systolic BP reduction in participants with IDH at baseline = b_treatment arm + (b_treatment arm:delta × 5) + b_treatment arm:IDH + (b_treatment arm:delta:IDH × 5) - b_comparator arm:IDH - (b_comparator arm:delta:IDH × 5)

Model #3: Standardized log hazard ratio per 5 mm Hg systolic blood pressure reduction in participants without IDH at baseline = b_treatment arm + (b_treatment arm:delta × 5)

**Figure S1: Effect of blood pressure lowering treatment on secondary outcomes, stratified by people with and without isolated diastolic hypertension at baseline.**

Forest plot shows the hazard ratios (HR) and 95% confidence intervals (CI) per 5 mmHg systolic blood pressure reduction, separately for each outcome

**
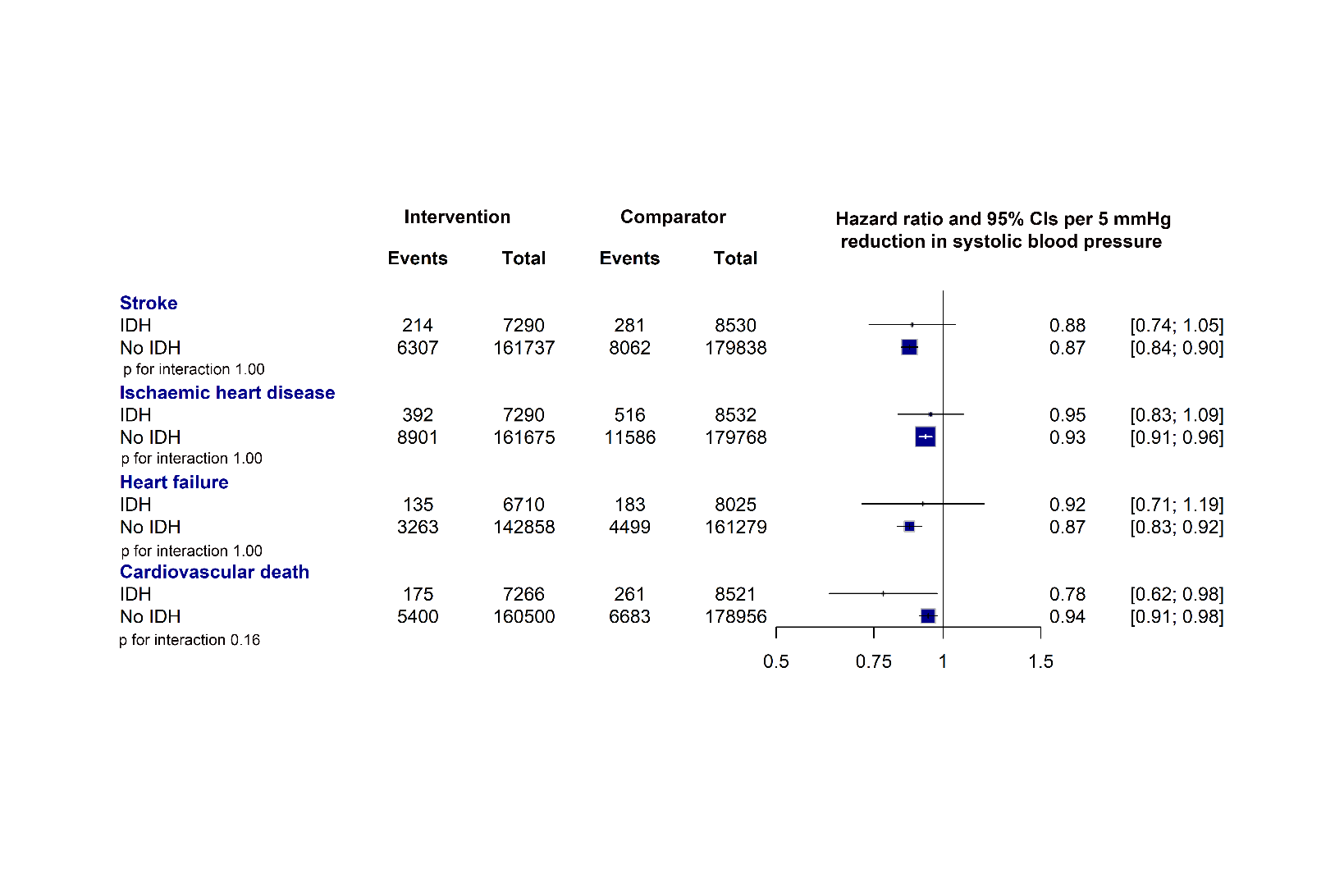
**

**Figure S2: Effect of blood pressure lowering treatment on major cardiovascular events and deaths, stratified by pre-existing cardiovascular status, in individual with isolated diastolic hypertension.**

Forest plot shows the hazard ratios (HR) and 95% confidence intervals (CI) per 5 mmHg systolic blood pressure reduction, separately for each outcome


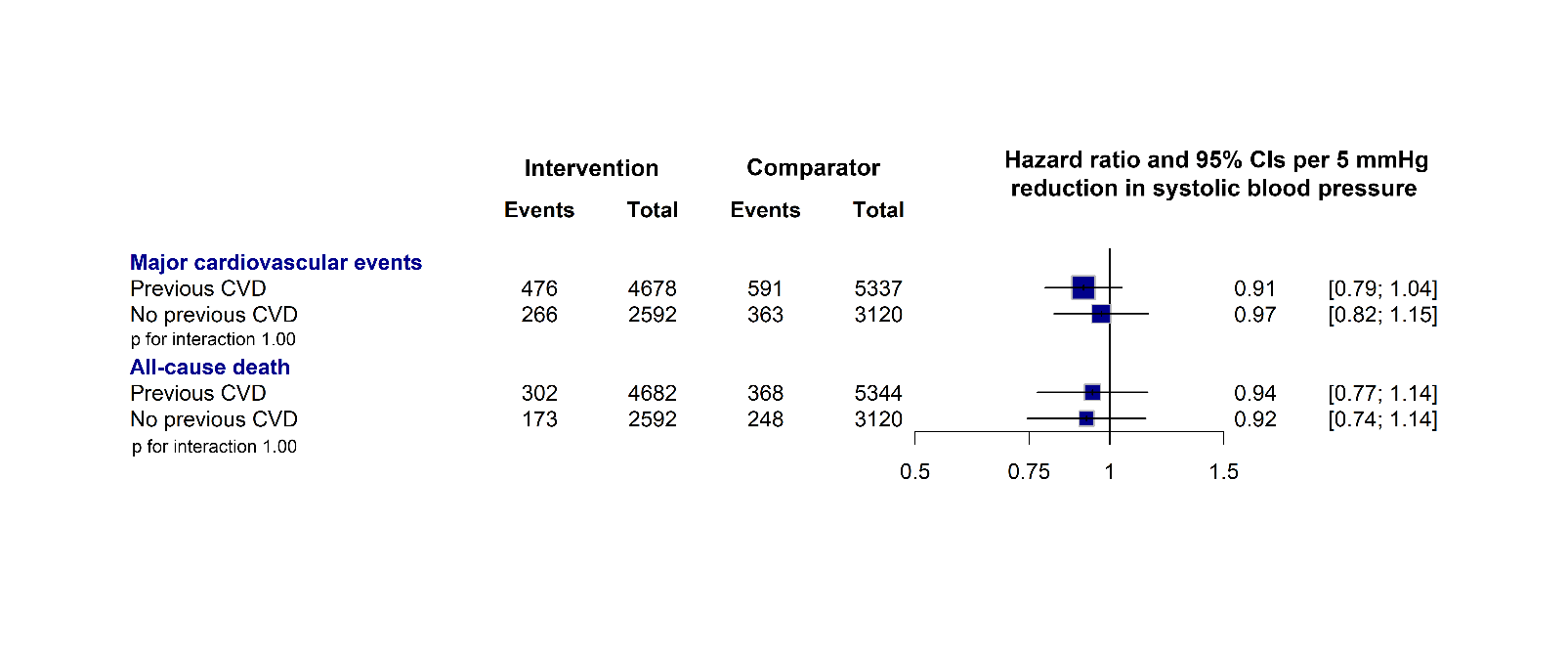


**Figure S3: Effect of blood pressure lowering treatment on major cardiovascular events, stratified by age categories at baseline, for isolated diastolic hypertension.**

Forest plot shows the hazard ratios (HR) and 95% confidence intervals (CI) per 5 mmHg systolic blood pressure reduction

**
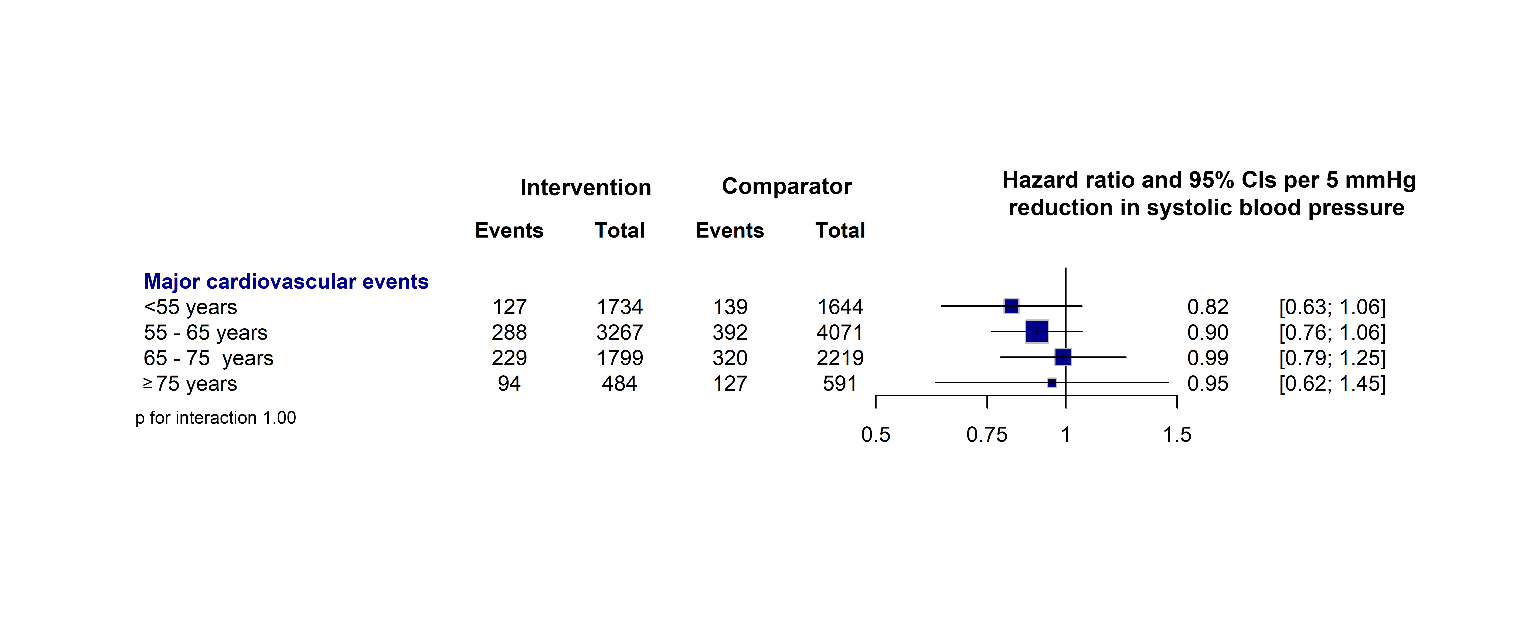
**

**Figure S4: Effect of blood pressure lowering treatment on major cardiovascular events and deaths, stratified by using antihypertensive drugs at baseline** **in people with isolated diastolic hypertension.**

Forest plot shows the hazard ratios (HR) and 95% confidence intervals (CI) per 5 mmHg systolic blood pressure reduction

**
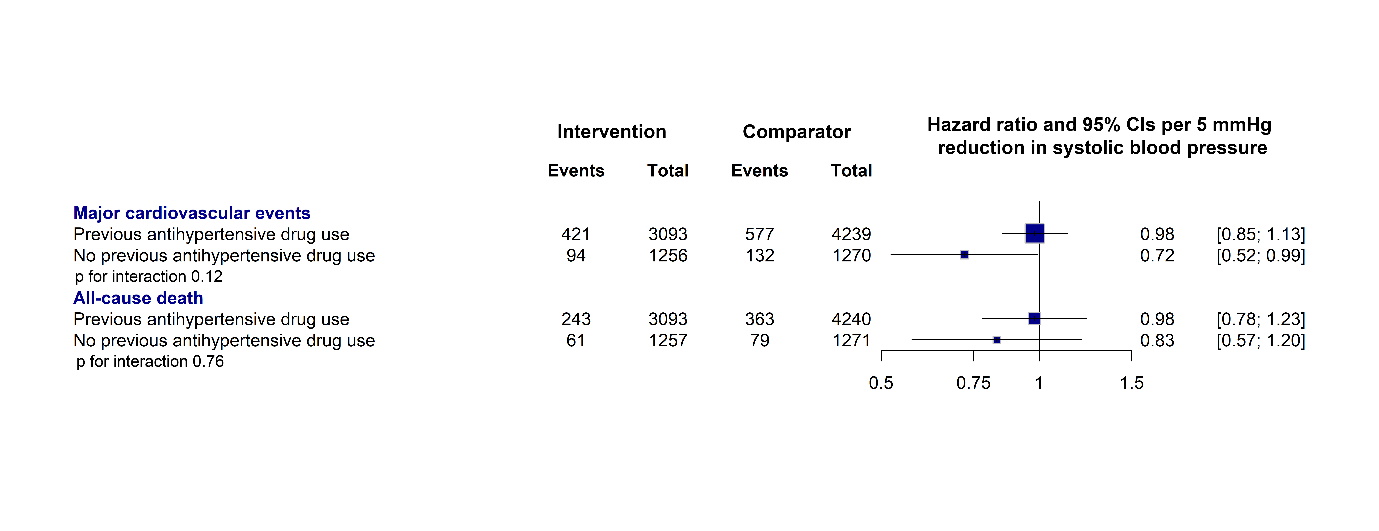
**

**Figure S5: Effect of blood pressure lowering treatment on major cardiovascular events and deaths, in people with and without isolated diastolic hypertension at baseline in trials that used automated devices for blood pressure measurements, for isolated diastolic hypertension.**

Forest plot shows the hazard ratios (HR) and 95% confidence intervals (CI) per 5 mmHg systolic blood pressure reduction, separately for each outcome

**
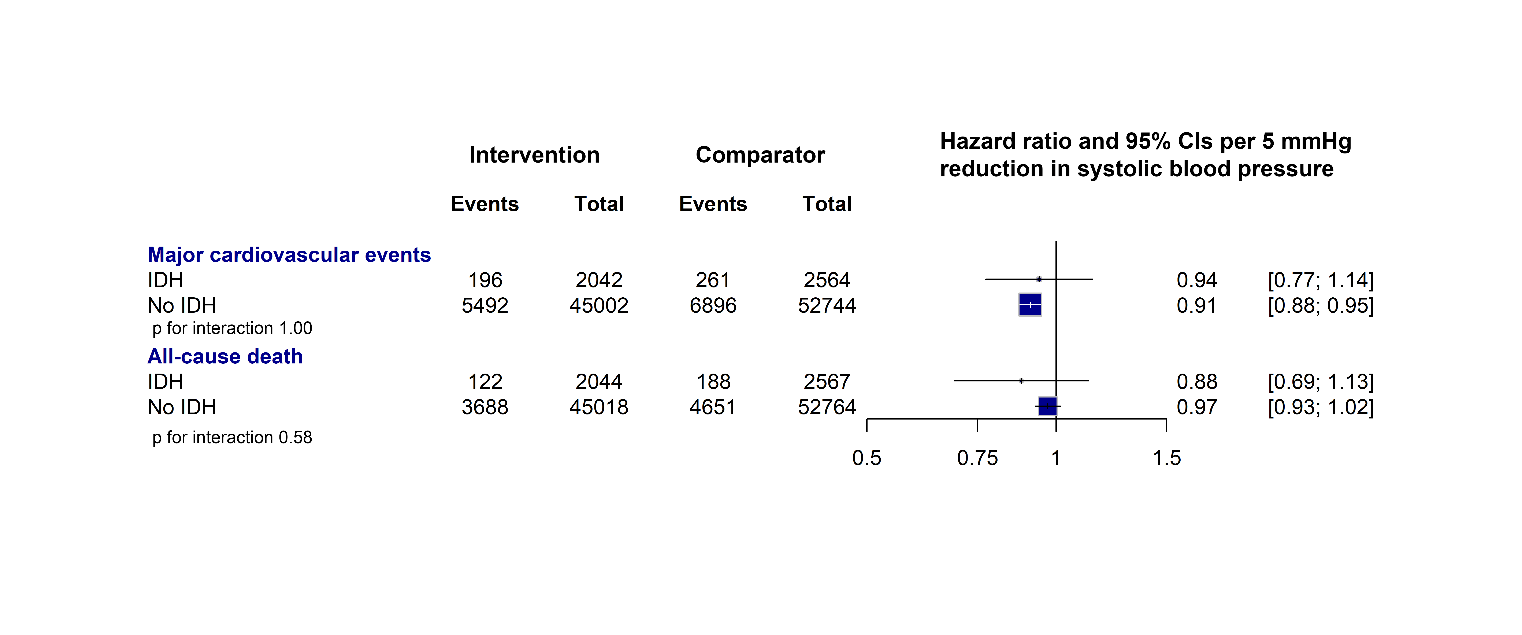
**

**Figure S6: Effect of blood pressure lowering treatment on major cardiovascular events and deaths, in people with and without isolated diastolic hypertension at baseline in trials that used manual devices for blood pressure measurements, for isolated diastolic hypertension.**

Forest plot shows the hazard ratios (HR) and 95% confidence intervals (CI) per 5 mmHg systolic blood pressure reduction, separately for each outcome


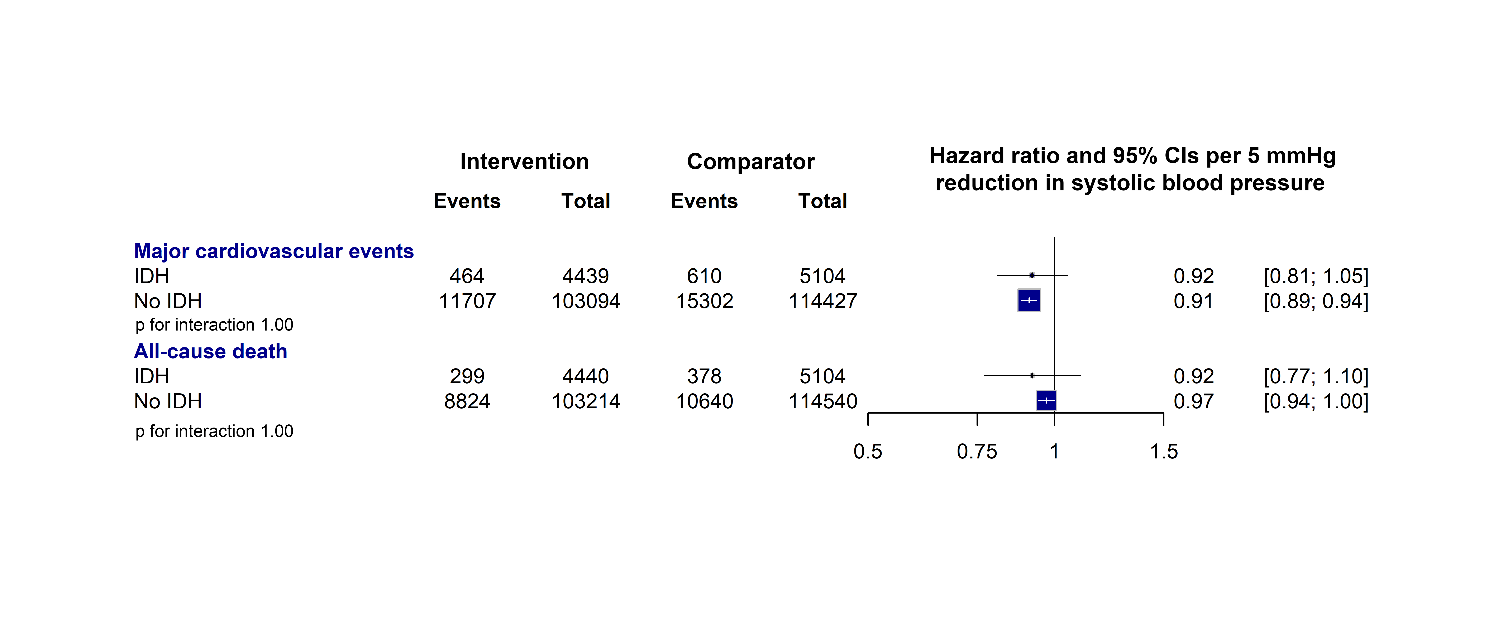


| **TableS1. Baseline Characteristics of Participants Stratified by with and without Isolated diastolic hypertension using an alternative IDH definition (SBP <140 mm Hg, DBP ≥ 90 mm Hg)** | | |
| --- | --- | --- |
| **Characteristics** | **With Isolated Diastolic Hypertension**  **(12,380)** | **Without Isolated Diastolic Hypertension**  **(345,890)** |
| Age | 57.0 (10.7) | 65.3 (9.5) |
| Gender | 4,046 (32.7) | 145,010 (41.9) |
| Systolic BP, mm Hg | 132.3 (5.5) | 153.1 (21.2) |
| Diastolic BP, mm Hg | 93.5 (4.0) | 87.1 (12.5) |
| Body mass index, kg/m^2^ | 28.8 (5.3) | 27.9 (7.4) |
| Comorbidity |  |  |
| Peripheral vascular disease | 248 (6.7) | 12,642 (9.7) |
| Atrial fibrillation | 551 (4.5) | 9,928 (2.9) |
| Diabetes | 2566 (20.8) | 98,948 (28.7) |
| Chronic kidney disease | 1674 (17.5) | 57467 (20.9) |
| Cerebrovascular disease | 1,845 (18.2) | 48,948 (17.6) |
| Ischemic heart disease | 3,808 (30.9) | 116,240 (33.7) |
| Previous use of non-trial medications (n, %) |  |  |
| Diuretic | 1,093 (21.6) | 33,311 (20.7) |
| α-blocker | 137 (3.9) | 4,707 (4.1) |
| β-blocker | 1,945 (37.8) | 57,958 (34.1) |
| ACE inhibitor | 1,453 (32.7) | 46,877 (32.2) |
| Angiotensin II receptor blocker | 136 (4.6) | 8442 (8.6) |
| Calcium channel blocker | 1,527 (29.7) | 54,466 (32.1) |
| Any BP-lowering drug | 5, 158 (61.1) | 149,008 (69.2) |
| Antiplatelet | 1,462 (49.2) | 49,530 (43.1) |
| Anticoagulant | 260 (10.4) | 6,305 (7.6) |
| Lipid-lowering treatment | 1,585 (39.8) | 52,857 (36.7) |
| Data are n (%) or mean (SD). ACE indicates angiotensin-converting enzyme, BP: blood pressure. | | |

**Figure S7: Effect of blood pressure lowering treatment on major cardiovascular events and secondary outcomes, stratified by people with and without isolated diastolic hypertension at baseline, using an alternative IDH definition (SBP <140 mm Hg, DBP ≥ 90 mm Hg).**

Forest plot shows the hazard ratios (HR) and 95% confidence intervals (CI) per 5 mmHg systolic blood pressure reduction, separately for each outcome


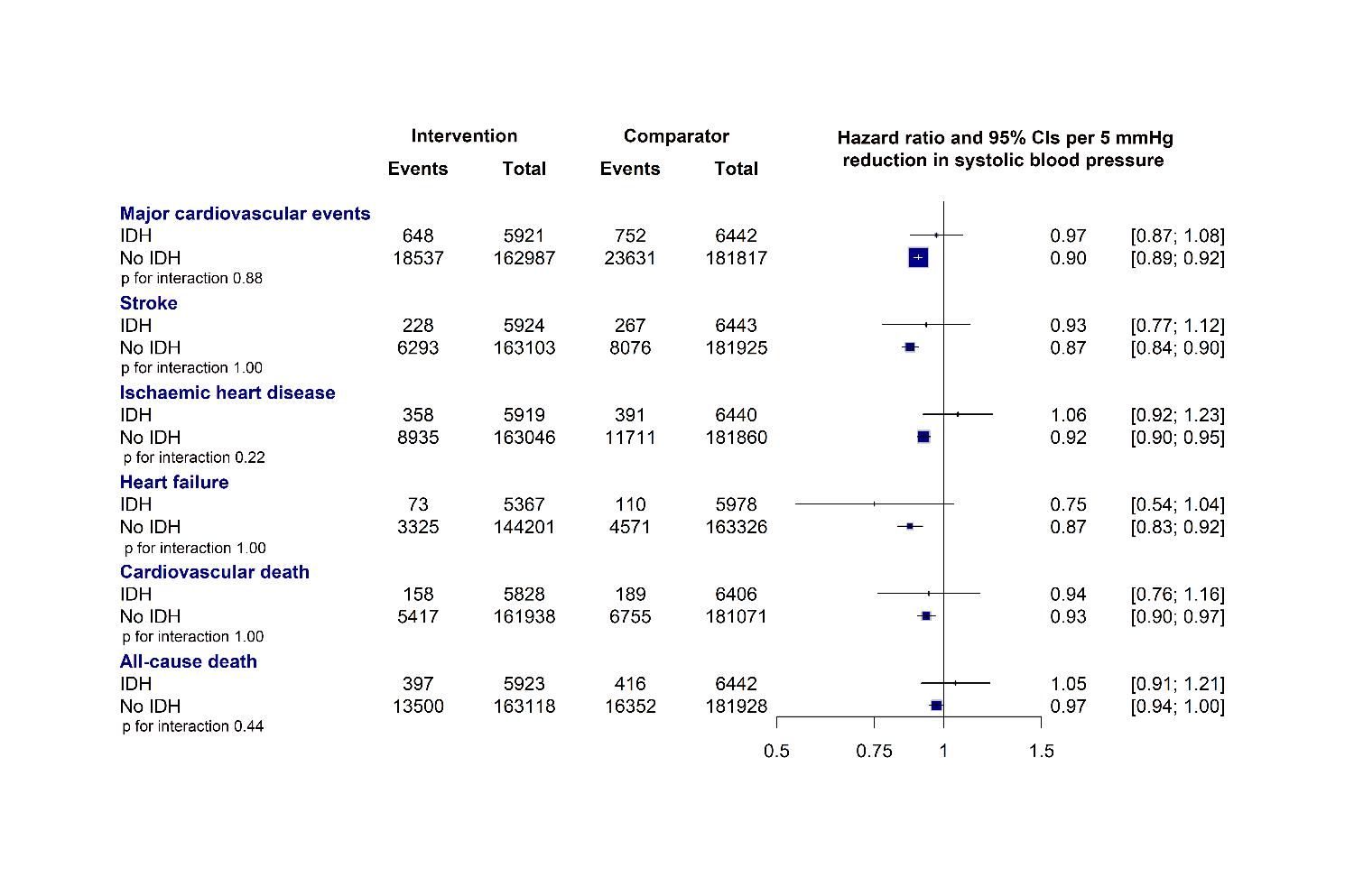


**Figure S8:** **Effect of blood pressure lowering treatment on major cardiovascular events, stratified by diastolic blood pressure categories at baseline, for people with systolic blood pressure of <140 mm Hg at baseline.**

Forest plot shows the hazard ratios (HR) and 95% confidence intervals (CI) per 5 mmHg systolic blood pressure reduction


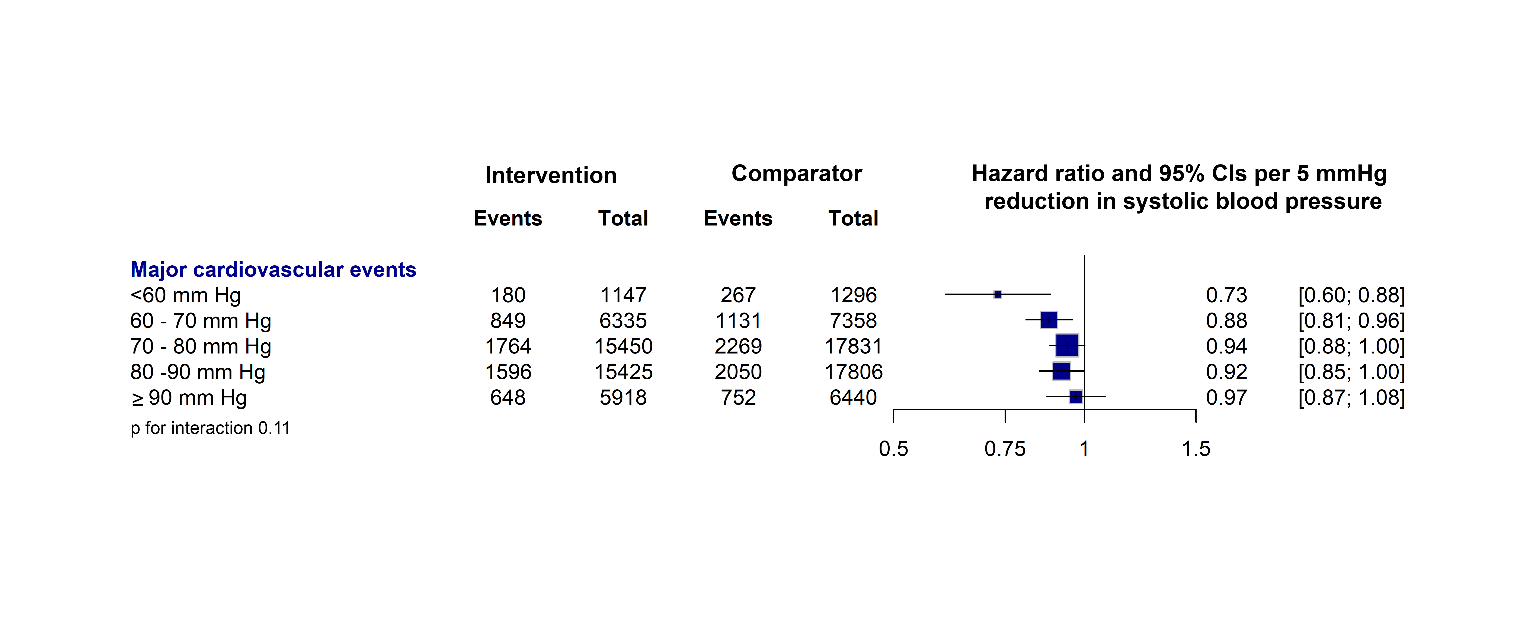


**Figure S9: Effect of blood pressure lowering treatment on major cardiovascular events and deaths, stratified by pre-existing cardiovascular status, for isolated diastolic hypertension, using an alternative IDH definition (SBP <140 mm Hg, DBP ≥ 90 mm Hg).**

Forest plot shows the hazard ratios (HR) and 95% confidence intervals (CI) per 5 mmHg systolic blood pressure reduction, separately for each outcome


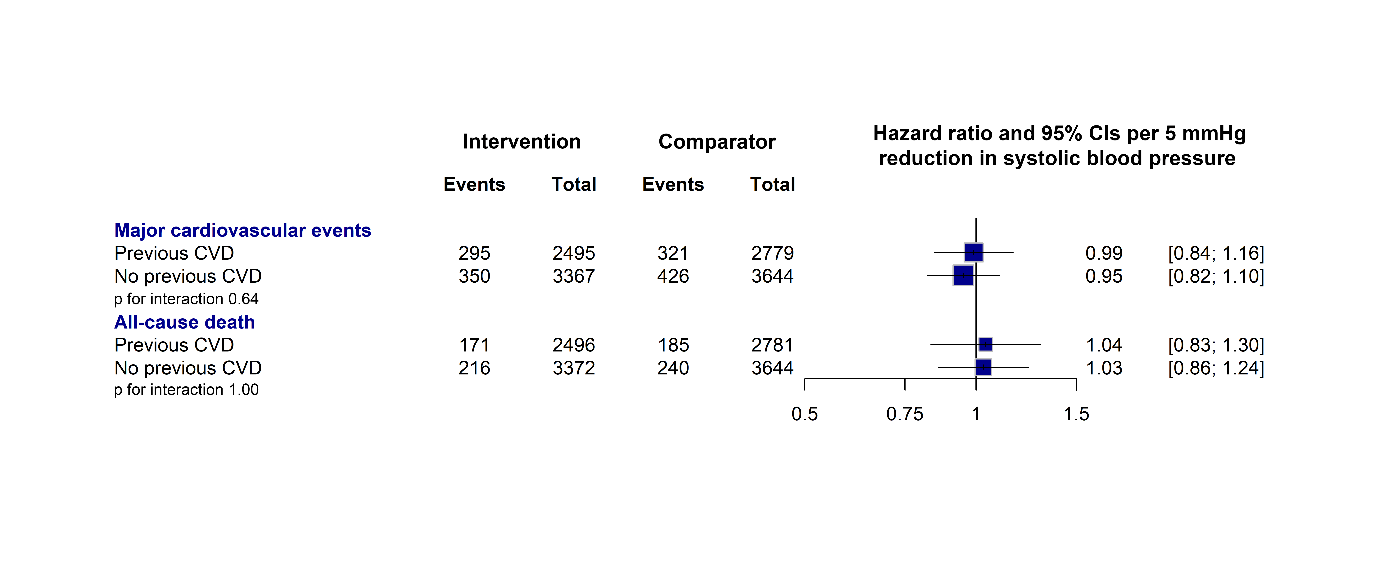


**Figure S10: Effect of blood pressure lowering treatment on major cardiovascular events, stratified by age categories at baseline, for isolated diastolic hypertension, using an alternative IDH definition (SBP <140 mm Hg, DBP ≥ 90 mm Hg).**

Forest plot shows the hazard ratios (HR) and 95% confidence intervals (CI) per 5 mmHg systolic blood pressure reduction


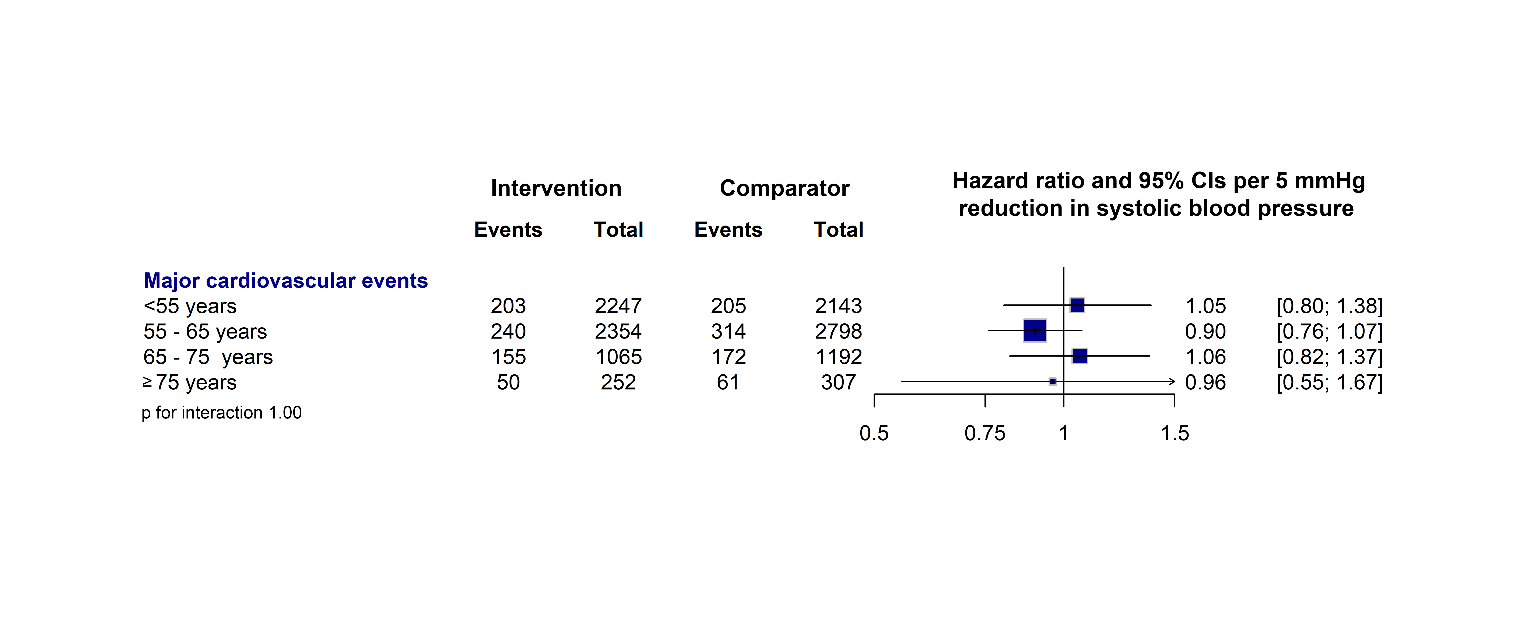


**Figure S11: Effect of blood pressure lowering treatment on major cardiovascular events and deaths, stratified by using antihypertensive drugs at baseline, for isolated diastolic hypertension, using an alternative IDH definition (SBP <140 mm Hg, DBP ≥ 90 mm Hg).**

Forest plot shows the hazard ratios (HR) and 95% confidence intervals (CI) per 5 mmHg systolic blood pressure reduction


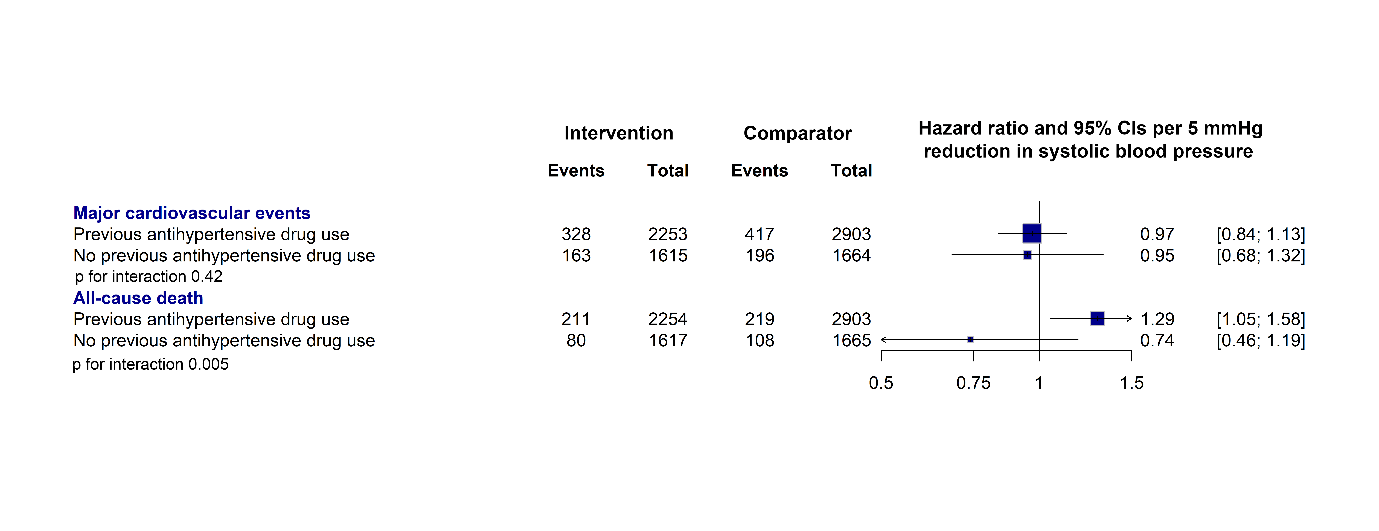


**Figure S12: Effect of blood pressure lowering treatment on major cardiovascular events and deaths, in people with and without isolated diastolic hypertension at baseline in trials that used automated devices for blood pressure measurements, for isolated diastolic hypertension, using an alternative IDH definition (SBP <140 mm Hg, DBP ≥ 90 mm Hg).**

Forest plot shows the hazard ratios (HR) and 95% confidence intervals (CI) per 5 mmHg systolic blood pressure reduction, separately for each outcome


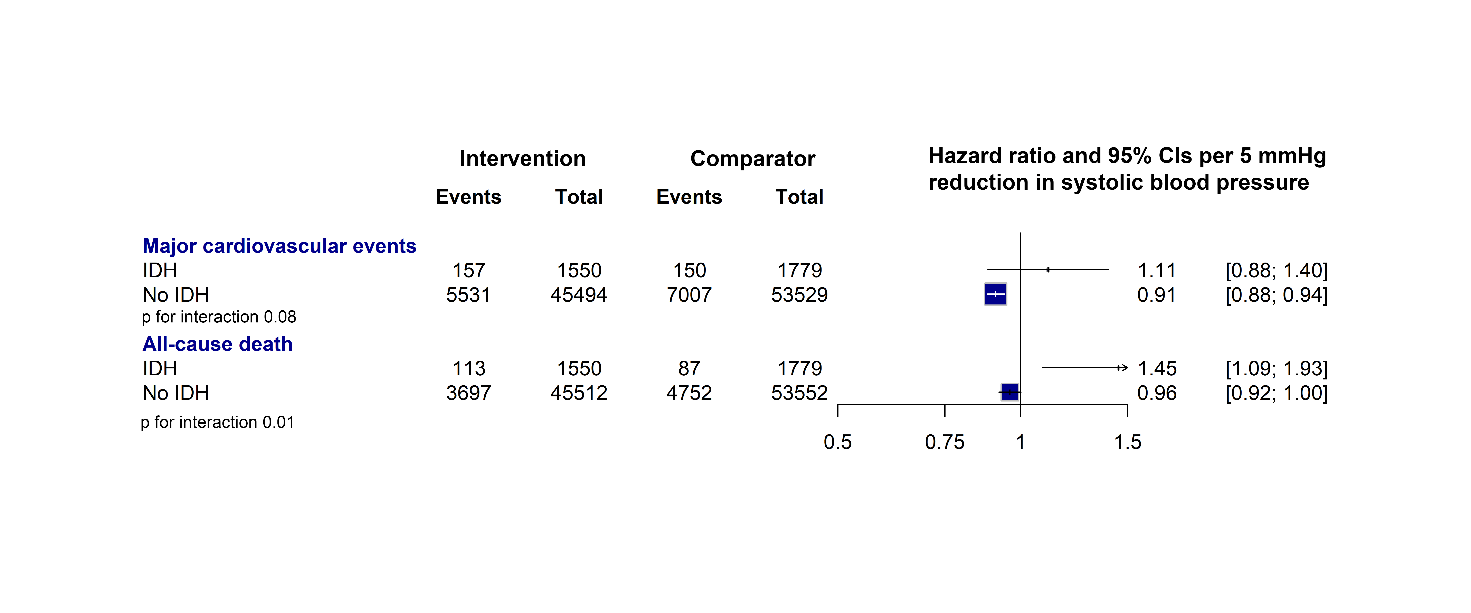


**Figure S13: Effect of blood pressure lowering treatment on major cardiovascular events and deaths, in people with and without isolated diastolic hypertension at baseline in trials that used manual devices for blood pressure measurements, for isolated diastolic hypertension, using an alternative IDH definition (SBP <140 mm Hg, DBP ≥ 90 mm Hg).**

Forest plot shows the hazard ratios (HR) and 95% confidence intervals (CI) per 5 mmHg systolic blood pressure reduction, separately for each outcome

**
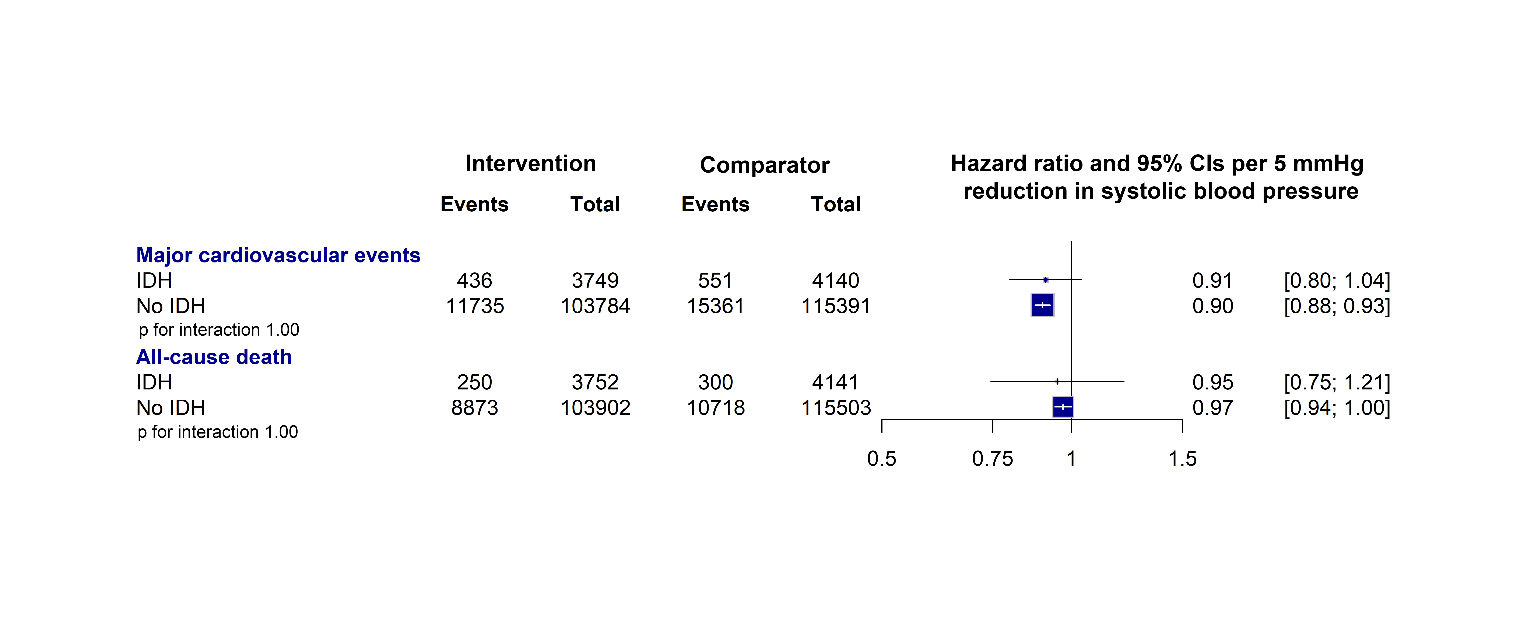
**

**Figure S14:** **Sensitivity analysis for the effect of blood pressure lowering treatment on major cardiovascular events and deaths, stratified by people with and without isolated diastolic hypertension at baseline, excluding the trials with risk of bias.**

Forest plot shows the hazard ratios (HR) and 95% confidence intervals (CI) per 5 mmHg systolic blood pressure reduction, separately for each outcome

**
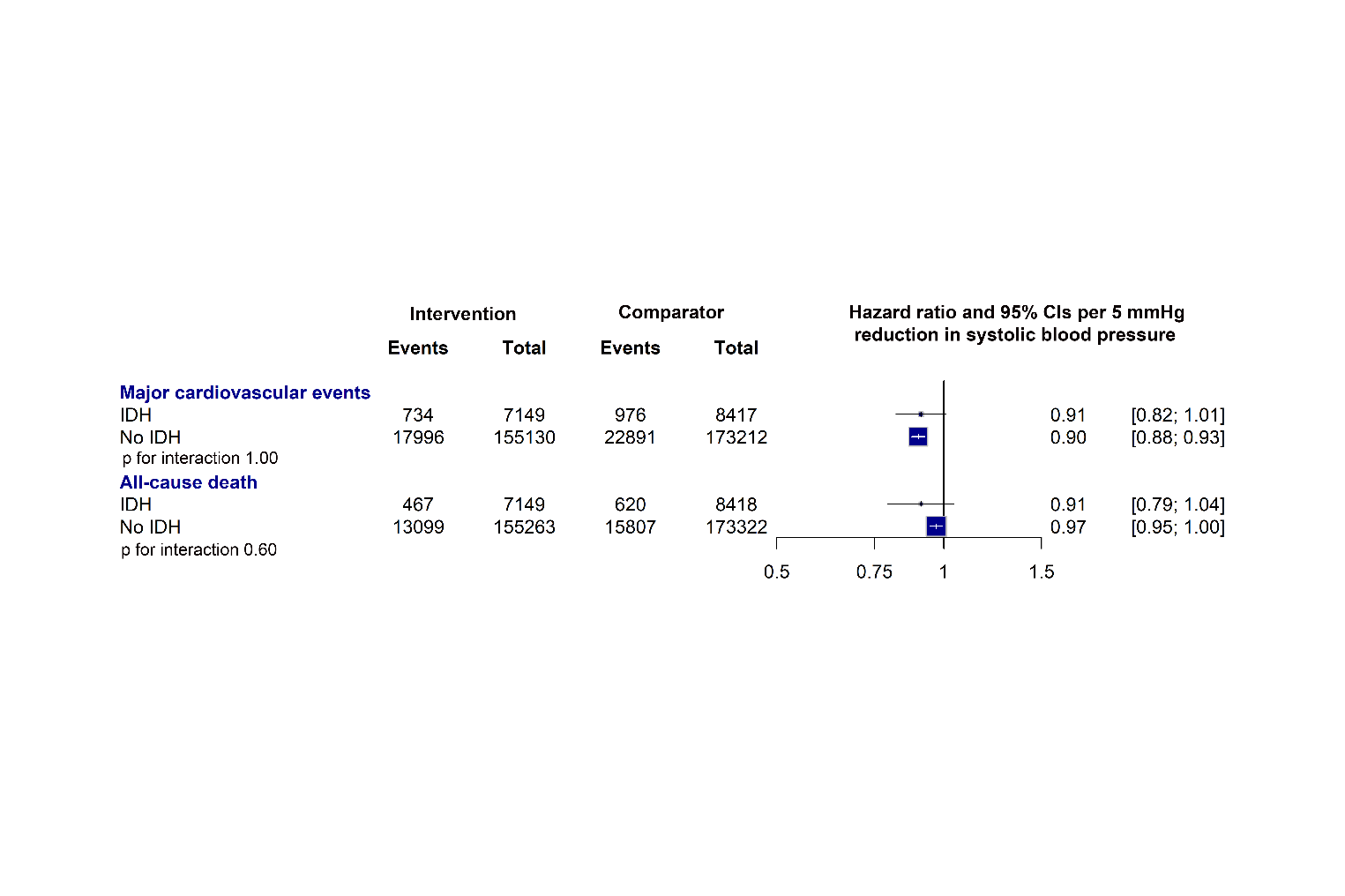
**

**Table 2: Effect of blood pressure lowering treatment on major cardiovascular events and deaths, stratified by people with and without isolated diastolic hypertension at baseline. Table shows the hazard ratios (HR) and 95% confidence intervals (CI) per 3 mmHg reduction in diastolic blood pressure**

| **Major cardiovascular disease** | **Intervention** | | **Comparator** | | **HR (0.95 CI)** |
| --- | --- | --- | --- | --- | --- |
|  | Events | Total | Events | Total |  |
| IDH | 738 | 7284 | 978 | 8525 | 0.94 (0.84 -1.06) |
| No IDH | 18419 | 161534 | 23371 | 179621 | 0.91 (0.86 – 0.93) |
| P for interaction = 1.00 | | | | | |
| **All-cause death** | | | | | |
| IDH | 478 | 7287 | 622 | 8528 | 0.94 (0.80 -1.10) |
| No IDH | 13396 | 161669 | 16116 | 179754 | 0.95 (0.92 – 0.97) |
| P for interaction = 0.85 | | | | | |

**Table 3: Effect of blood pressure lowering treatment on major cardiovascular events, stratified by diastolic blood pressure categories at baseline, for people with systolic blood pressure of <130 mm Hg at baseline.**

Table shows the hazard ratios (HR) and 95% confidence intervals (CI) per 3 mmHg reduction in diastolic blood pressure

| **Major cardiovascular disease** | **Intervention** | | **Comparator** | | **HR (0.95 CI)** |
| --- | --- | --- | --- | --- | --- |
|  | **Events** | **Total** | **Events** | **Total** |  |
| <60 mm Hg | 133 | 916 | 203 | 1012 | 0.68 (0.52 -0.89) |
| 60-70 mm Hg | 588 | 4573 | 772 | 5279 | 0.89 (0.77 – 1.02) |
| 70-80 mm Hg | 961 | 9008 | 1276 | 10228 | 0.93 (0.82 -1.05) |
| 80-90 mm Hg | 598 | 6062 | 811 | 7097 | 0.94 (0.81 -1.08) |
| > 90 mm Hg | 140 | 1222 | 167 | 1428 | 1.00 (0.80 – 1.27) |
| P for interaction = 0.26 | | | | | |

**Table 4: Effect of blood pressure lowering treatment on major cardiovascular events and deaths, stratified by people with and without isolated diastolic hypertension at baseline, using an alternative IDH definition (SBP <140 mm Hg, DBP ≥ 90 mm Hg).**

Table shows the hazard ratios (HR) and 95% confidence intervals (CI) per 3 mmHg reduction in diastolic blood pressure

| **Major cardiovascular disease** | **Intervention** | | **Comparator** | | **HR (0.95 CI)** |
| --- | --- | --- | --- | --- | --- |
|  | **Events** | **Total** | **Events** | **Total** |  |
| IDH | 648 | 5918 | 752 | 6440 | 0.96 (0.87 -1.07) |
| No IDH | 18509 | 162900 | 23597 | 181706 | 0.91 (0.88 – 0.93) |
| P for interaction = 0.88 | | | | | |
| **All-cause death** | | | | | |
| IDH | 397 | 5921 | 418 | 6441 | 1.00 (0.87-1.17) |
| No IDH | 13472 | 163035 | 16322 | 181841 | 0.94 (0.92 -0.97) |
| P for interaction = 0.44 | | | | | |

**Table 5: Effect of blood pressure lowering treatment on major cardiovascular events, stratified by diastolic blood pressure categories at baseline, for people with systolic blood pressure of <140 mm Hg at baseline.**

Table shows the hazard ratios (HR) and 95% confidence intervals (CI) per 3 mmHg reduction in diastolic blood pressure

| **Major cardiovascular disease** | **Intervention** | | **Comparator** | | **HR (0.95 CI)** |
| --- | --- | --- | --- | --- | --- |
|  | **Events** | **Total** | **Events** | **Total** |  |
| < 60 mm Hg | 180 | 1147 | 267 | 1296 | 0.74 (0.59 -0.92) |
| 60-70 mm Hg | 849 | 6335 | 1131 | 7358 | 0.85 (0.75 – 0.95) |
| 70-80 mm Hg | 1764 | 15450 | 2269 | 17831 | 0.90 (0.82 -0.98) |
| 80-90 mm Hg | 1596 | 15425 | 2050 | 17806 | 0.92 (0.84 -1.01) |
| > 90 mm Hg | 648 | 5918 | 752 | 6440 | 0.97 (0.87 – 1.08) |
| P for interaction = 0.11 | | | | | |

1. Law MR, Morris JK, Wald NJ. Use of blood pressure lowering drugs in the prevention of cardiovascular disease: meta-analysis of 147 randomised trials in the context of expectations from prospective epidemiological studies. *Bmj* 2009;**338**:b1665. doi: <https://doi.org/10.1136/bmj.b1665>

2. Thomopoulos C, Parati G, Zanchetti A. Effects of blood pressure lowering on outcome incidence in hypertension. 1. Overview, meta-analyses, and meta-regression analyses of randomized trials. *J Hypertens* 2014;**32**:2285-2295. doi: <https://doi.org/10.1097/hjh.0000000000000378>

3. Canoy D, Copland E, Nazarzadeh M, Ramakrishnan R, Pinho-Gomes AC, Salam A, et al. Antihypertensive drug effects on long-term blood pressure: an individual-level data meta-analysis of randomised clinical trials. *Heart* 2022;**108**:1281-1289. doi: <https://doi.org/10.1136/heartjnl-2021-320171>
